# Loss of CsrA in *Acinetobacter baumannii* leads to lethal dysregulation of alanine metabolism

**DOI:** 10.1073/pnas.2624702123

**Published:** 2026-09-29

**Authors:** Rosa L. Sava, Michael J. Gebhardt

**Affiliations:** ^a^ Department of Microbiology and Immunology, University of Iowa, Iowa City, IA 52242

**Keywords:** *Acinetobacter baumannii*, CsrA, posttranscriptional regulation, alanine metabolism

## Abstract

Posttranscriptional regulation is a key mechanism by which bacteria coordinate gene expression in response to changing environments. In the human pathogen *Acinetobacter baumannii*, the importance of posttranscriptional gene expression control is exemplified by CsrA, a highly conserved posttranscriptional regulator that is essential for viability in multiple settings, ranging from growth in rich laboratory media to infection-relevant conditions like human serum. However, *csrA* is dispensable for growth in chemically defined media with a single carbon source. We leveraged this condition-specific essentiality to identify suppressor mutations that regained the ability to grow in rich media. These analyses identified Lrp, a transcription factor that regulates hundreds of genes in *A. baumannii.* Among the Lrp regulon, we identified a four gene locus involved with uptake and utilization of D-alanine. We determined that two transcripts encoded by this locus, *dadA* and *cycA2*, are repressed by CsrA. The absence of *csrA* leads to overexpression of both *dadA* and *cycA2*, which is toxic, even in wild type *A. baumannii*. In the *csrA* mutant, this dysregulation contributes to impaired cell envelope integrity and increased sensitivity to several clinically relevant cell-wall targeting antibiotics, including carbapenems. Collectively, our data indicate that Lrp and CsrA coordinately balance expression of D-alanine utilization genes in *A. baumannii*, at the transcriptional and posttranscriptional levels, respectively. Moreover, it appears that a key aspect of CsrA’s essentiality in *A. baumannii* is to counter the activities of Lrp, a second global regulator. These findings further highlight the complex interplay between transcriptional and posttranscriptional regulators in bacteria.

## Significance

In the pathogen *Acinetobacter baumannii*, the posttranscriptional regulator CsrA is required for growth under certain environmental conditions, including rich growth media and infection-relevant conditions like human serum. However, CsrA is dispensable for *A. baumannii* grown under nutrient limited conditions, such as a minimal medium containing a single carbon source. Using classical genetic approaches to explore this conditional essentiality, we found that CsrA and Lrp, a global transcription regulator, coordinate expression of several genes involved with D-alanine metabolism. Consistent with D-alanine’s importance to peptidoglycan biosynthesis, *csrA* mutants exhibit impaired cell envelope integrity. Our findings provide a potential mechanism for the essentiality of CsrA in *A. baumannii* and further highlight how global regulatory networks intersect to promote fitness under changing environmental conditions.

*Acinetobacter baumannii* is a Gram-negative bacterium that is evolving into a significant human pathogen. *A. baumannii* infections, which can occur in a variety of tissues including the lung, blood stream, and urinary tract, are opportunistic in nature, most frequently occurring in hospitalized or immunocompromised individuals (1, 2). Infections caused by the organism are especially concerning due to high rates of antibiotic resistance among contemporary clinical isolates. Indeed, antibiotic resistant *A. baumannii* has been identified as an organism of highest concern by the CDC and WHO (3–5). In addition, *A. baumannii* clinical isolates frequently exhibit desiccation resistance and can survive both routine decontamination practices and periods of nutrient limitation in the clinical setting (6, 7). These emerging antibiotic resistance and stress-resistance phenotypes; referred to as a “persist-and-resist” strategy, when coupled with a trend toward increased virulence potential among currently circulating isolates, has undoubtedly contributed to the rise of *A. baumannii* as a problematic pathogen (8–10).

Bacterial survival hinges on the ability to sense and respond properly to fluctuations in the environment. These responses are largely mediated through alterations in gene expression (11) and occur at both the transcriptional and posttranscriptional level. The bacterial posttranscriptional response is frequently mediated by regulatory RNA binding proteins (RBPs) whose function drives requisite changes in gene expression (11, 12). In *A. baumannii,* previous works demonstrate that CsrA, a highly conserved bacterial RBP, plays an important role in the response to many of the persist-and-resist phenotypes mentioned above, including desiccation resistance, biofilm formation, and metabolic versatility (13–15). Proteins in the CsrA family–first named for its role in carbon storage regulation in *Escherichia coli*–are found across the bacterial kingdom and exert posttranscriptional control over gene expression by directly associating with target mRNA transcripts, often influencing the translation and/or stability of the target RNA (16–19). In *A. baumannii*, the importance of CsrA is highlighted by the observation that CsrA is essential for growth in rich media (13–15). Furthermore, *A. baumannii* mutants lacking *csrA* are impaired for growth in the presence of certain amino acids in the growth medium, including alanine and arginine (14, 15). The viability defects associated with *csrA* also extend into infection relevant conditions, where *A. baumannii csrA* mutants exhibit impaired growth in the *Galleria mellonella* larval infection model, human urine, and human serum (13–15). In line with a role in virulence, a recent study by Singh and colleagues demonstrates that CsrA also impacts a phenotypic switch between virulent and avirulent growth states (20).

Given the important role of CsrA in regulating *A. baumannii* physiology and survival in diverse environmental conditions, we sought to identify the mechanisms underlying the conditional essentiality of *csrA*. Using genetic analyses, we determined that the transcription factor Lrp, which we show is a global regulator in *A. baumannii*, contributes to suppression of *csrA* phenotypes. Lrp is a member of the “feast/famine regulatory proteins” family, which are found across the bacterial kingdom and primarily act as transcription regulators (21). We further demonstrate that, in conjunction with CsrA, Lrp coordinates expression of a suite of genes involved with D-alanine metabolism, including a D-amino acid dehydrogenase, an alanine racemase, and a D-amino acid permease. Lrp positively regulates the transcription of these genes, whereas CsrA represses their translation. As D-alanine is a key building block required for the synthesis of peptidoglycan (PG) and the bacterial cell wall, we also examined what role CsrA may play on cell envelope homeostasis and determined that cells lacking *csrA* have altered sensitivities to several antimicrobial compounds targeting cell wall biosynthesis. Moreover, ectopic expression of the CsrA-regulated D-alanine genes causes growth arrest in wild-type *A. baumannii* cells and impart an antibiotic resistance profile similar to the ∆*csrA* strain. These findings suggest that at least one aspect of the *csrA* phenotype results from dysregulation of the D-amino acid pool in the cell. We propose a model whereby two global regulators—Lrp and CsrA—coordinately regulate D-alanine availability in *A. baumannii*, and so doing, contribute to cell wall homeostasis in this important human pathogen.

## Results

### Isolation of ∆*csrA* Suppressors.

**Fig. 1.**
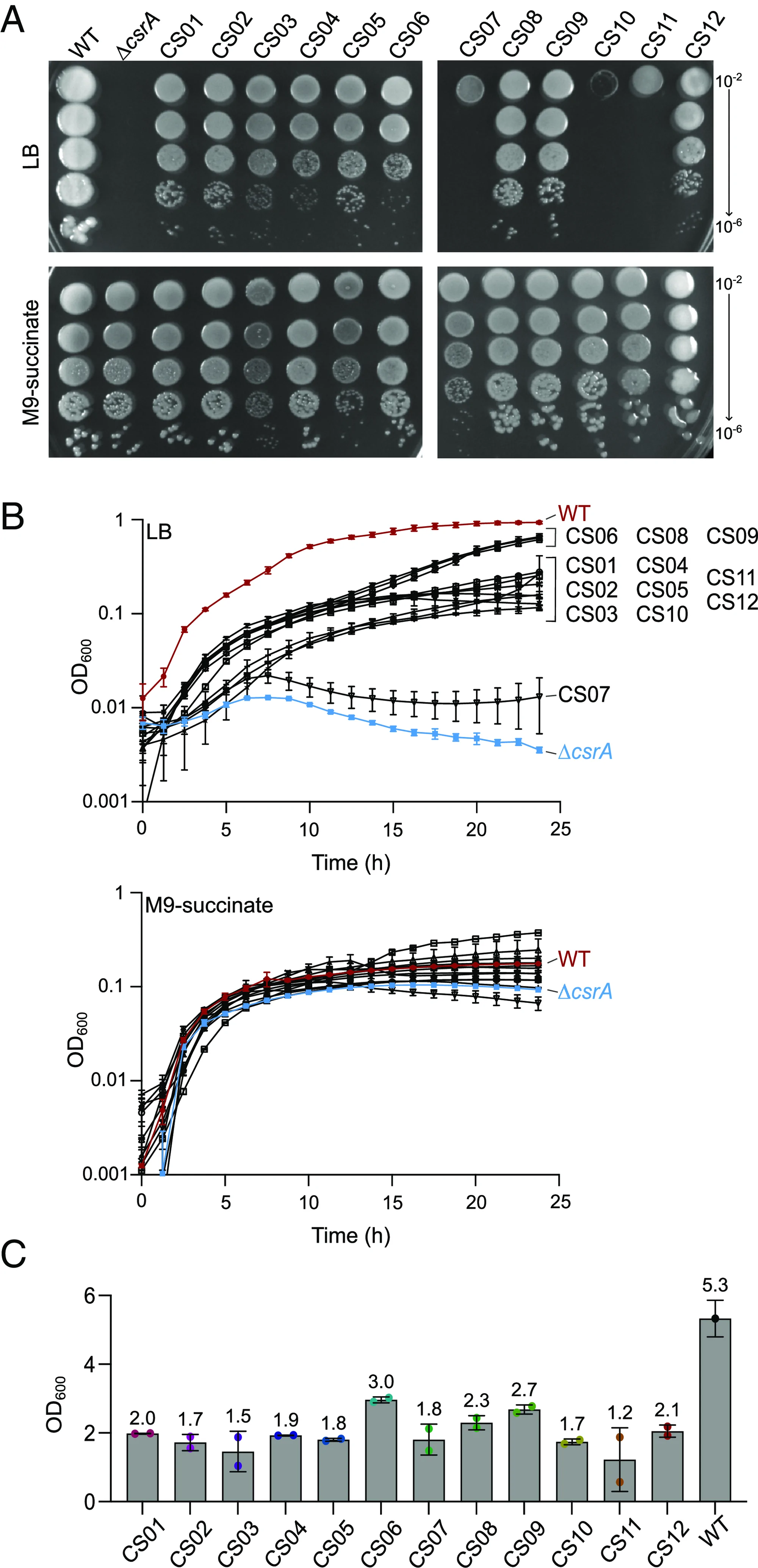
Isolation of ∆*csrA* suppressors. (*A*) The indicated strains, including wild-type AB5075 (WT), the isogenic *csrA* mutant (*∆csrA*), and the ∆*csrA* suppressors (CS) were grown overnight in M9-succinate medium, normalized by optical density, serially diluted, and plated on LB and M9-succinate agar plates and incubated overnight at 37 °C. Numbers at right indicate the dilution plated, with 10^0^ corresponding to an OD_600_ of 1.0. (*B*) Growth curves of indicated strains in LB (*Top*) or M9-succinate (*Bottom*). Data shown as the mean OD_600_ for biological triplicate wells; error bars indicate one SD of the mean. (*C*) Graph depicts final cell density (in OD_600_) of overnight cultures grown in LB for the indicated strains. Data points represent two biological replicates; bars indicate mean density and error bars represent one SD. Experiments were repeated at least three times on different days.

Consistent with previous reports, an isogenic strain carrying a deletion of *csrA* in the *A. baumannii* AB5075 strain failed to grow on rich media (i.e., LB) but grew nearly indistinguishably from wild-type cells in M9 minimal media with succinate as the sole carbon source (13). To extend this observation, we screened a panel of *A. baumannii* isolates using CRISPR interference (CRISPRi; 22, 23). Knock-down of *csrA* had little effect on M9-succinate but abrogated growth on LB in each strain tested (*SI Appendix,* Fig. S1). To gain insight into the genetic elements that underlie this phenotype, we isolated 12 independent and spontaneously occurring suppressor clones that exhibited restored growth in LB (hereafter referred to as CS for *csrA* Suppressor). The suppressors displayed varying growth patterns on LB with most of the mutants producing visibly smaller colonies than wild-type AB5075 (Fig. 1*A*). Interestingly, some of the suppressors (i.e., CS03, CS05, and CS07) produced smaller colonies when grown on M9-succinate, while others resembled WT (i.e., CS02 and CS08). When cells from overnight cultures were serially diluted and plated on LB, most grew to a similar dilution as wild type AB5075 (WT), although CS07, CS10, and CS11 grew poorly on solid LB. Consistent with the results observed on solid media, growth assays conducted in liquid LB showed that the *∆csrA* suppressors displayed altered growth kinetics and reached a lower terminal density than WT (Fig. 1 *B* and *C*). As expected, the suppressors exhibited WT growth kinetics when cultured in liquid M9-succinate, although some variations were observed (Fig. 1*B*).

The observed differences among the *∆csrA* suppressors with respect to restoring growth in LB suggests that multiple mutational pathways may suppress the ∆*csrA* phenotype. Whole genome sequencing (WGS) of the 12 suppressor strains supports this, as we detected large deletions (greater than 15 kb) in five suppressors and distinct single nucleotide polymorphisms in eight suppressors (Table 1 and Dataset S1). We did not detect mutations in three suppressors (CS06, CS09, and CS10); these strains may have rearrangements our analysis pipeline failed to detect.

**Table 1.** Summary of whole genome sequencing analysis

Strain	Detected mutations^*^
CS01	∆*csrA*, *lrp* region^∆3834546-3863318^, ABUW_RS09985^Y200M^ ABUW_RS18890^K528*^
CS02	∆*csrA*, *lrp* region^∆3846214-3863195^, *rpsL*^G92S^
CS03	*∆csrA, rplE^+D104,+R105+L106^*
CS04	∆*csrA*, *lrp* region^∆3853671-3879950^
CS05	∆*csrA*, *ettA^H217R^*
CS06	∆*csrA*
CS07	∆*csrA*, prophage 1 *phiOXA*^∆546153-581261^, ABUW_RS19720^fs-645-684*^, *ABUW_RS18890^G481V^*
CS08	∆*csrA, ABUW_RS20705^R179R^*
CS09	∆*csrA*
CS10	∆*csrA*
CS11	∆*csrA*, *ABUW_RS07280^S47Y^*
CS12	∆*csrA, lrp* region^∆3838548-3865742^, *bfmS^L500F^*

^*^indicates premature stop codon, fs indicates frameshift.

### Lrp Is a *csrA* Suppressor.

**Fig. 2.**
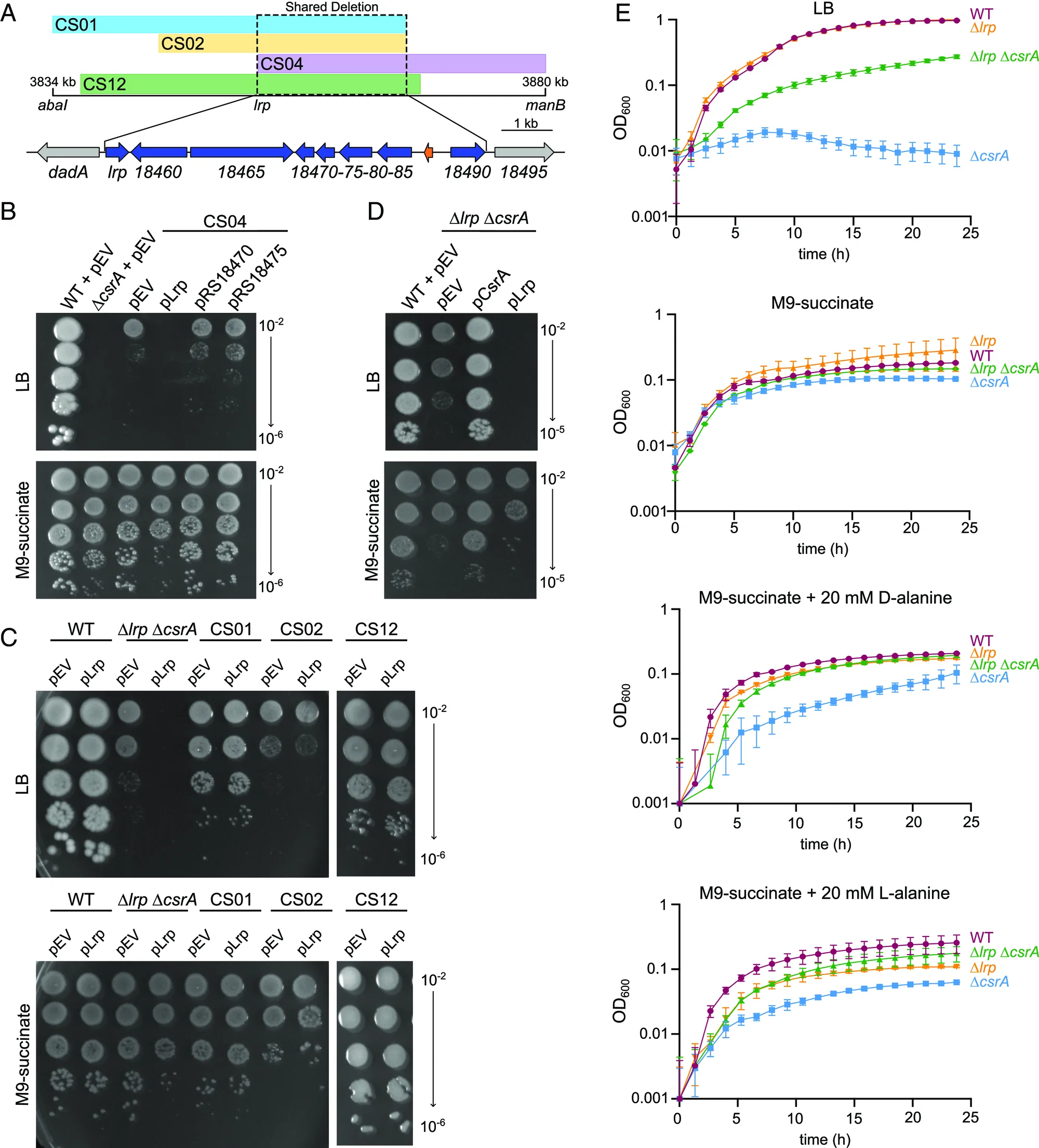
Lrp is a *csrA* suppressor. (*A*) Schematic depicts the *lrp* chromosomal locus. Shaded regions indicate the boundaries of the deletions detected in the indicated *csrA* suppressor strains: CS01 (blue), CS02 (yellow), CS04 (purple), and CS12 (green). The region shared among the four suppressors is depicted below, with blue and gray arrows indicating genes; sRNA106 is colored orange. Arrow orientation indicates the direction of transcription; numbers indicate locus tags (ref-seq annotations begin with the prefix *ABUW_RS*) for uncharacterized genes. (*B*–*D*) Plating analysis for the indicated strains harboring the indicated plasmids grown on LB or M9-succinate as indicated: (*B*), AB5075 (WT), ∆*csrA,* and suppressor CS04 harboring the indicated plasmids. (*C*) WT, ∆*lrp ∆csrA,* and suppressor strains CS01, CS02, and CS12 harboring the indicated plasmids. (*D*) WT and ∆*lrp ∆csrA* harboring the indicated plasmids. pEV, empty vector control; pLrp, *lrp* plasmid; pRS18470, *ABUW_RS18470* plasmid; pRS18475, *ABUW_RS18475* plasmid; pCsrA, *csrA* plasmid. (*E*) Growth curves of WT, ∆*csrA, ∆lrp,* and ∆*lrp ∆csrA* in LB or M9-succinate with or without 20 mM D- or L-alanine. Growth curves were repeated independently three times; data from a single representative run are presented as the mean OD_600_ for technical triplicate wells at the indicated time points. Error bars indicate one SD of the mean. Plating assays in panels *B*–*D* were conducted as described in the legend for Fig. 1. Numbers to the right indicate the dilution plated.

The deletions in CS01, CS02, CS04, and CS12 map to the same chromosomal region and share a total of eight genes (Fig. 2*A*), including *ABUW_RS18455*, encoding an Lrp family transcription regulator, *ABUW_RS18465*, encoding a predicted TonB-dependent siderophore receptor, *ABUW_RS18480,* encoding a predicted short-chain dehydrogenase, a noncoding RNA (sRNA106), and several hypothetical proteins (*ABUW_RS18470, ABUW_RS18475, ABUW_ RS18485*). To determine which of these genes is responsible for suppressing the ∆*csrA* growth phenotype, we introduced plasmid-borne copies of each gene into suppressor strain CS04. Intriguingly, strain CS04 harboring the *lrp* plasmid (pLrp) failed to grow on LB (Fig. 2*B*), while none of the other genes affected growth on LB (*SI Appendix,* Fig. S1). This finding implicates *lrp* as being responsible for the suppression phenotype in CS04. Interestingly, however, when we introduced pLrp into suppressors CS01, CS02, or CS12, the strains retained the ability to grow on LB (Fig. 2*C*), which suggests that the growth phenotype reversion observed for CS04 may be due to the regulatory effects of Lrp on a gene or genes that were lost in the larger deletions that gave rise to CS01, CS02, CS12.

To further examine Lrp as a possible suppressor of *csrA*, we deleted *lrp* from the AB5075 background (∆*lrp*), which grew similarly to WT in LB and M9-succinate (Fig. 2*E*). When we deleted *csrA* from the ∆*lrp* background, the resulting strain (i.e., ∆*lrp ∆csrA*) grew on both solid and liquid LB (Fig. 2 *D* and *E*). As with the isolated suppressors, the ∆*lrp* ∆*csrA* strain displayed reduced growth in liquid LB compared to WT (Fig. 2 *E*, *Top* panel) and introduction of pLrp into the ∆*lrp ∆csrA* strain background abrogated growth on LB agar (Fig. 2*D*). Together, the results obtained with the *∆lrp* strain largely mirrored the ∆*csrA* suppressors and reinforce that Lrp, or its regulatory effects, are linked with the ∆*csrA* growth phenotype.

### Lrp Is a Global Regulator in AB5075.

**Fig. 3.**
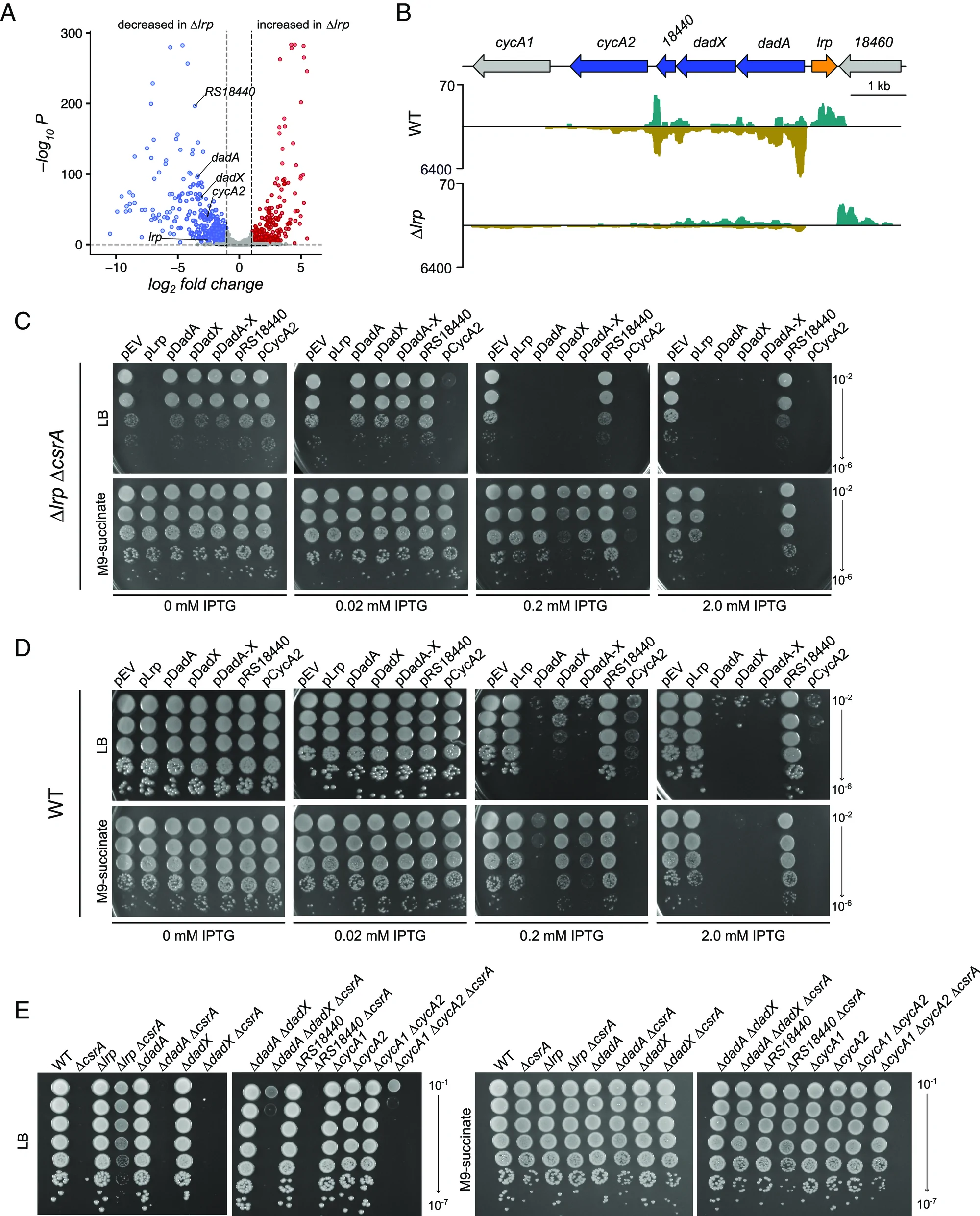
Lrp is a global transcription regulator in AB5075. (*A*) Volcano plot of RNA-seq comparing ∆*lrp* to WT. Blue dots depict genes with decreased transcript abundance in ∆*lrp*; red dots depict those with increased abundance in ∆*lrp*; gray points are not different than wild-type. Significance defined as a log_2_ fold change <1 or >1 and *q*-value < 0.05; genes in the *dad/lrp* locus are indicated. (*B*) Schematic of *dad* locus and corresponding normalized RNA-seq read depth from WT and ∆*lrp* mutant cells depicted below the annotated genes. Reads mapping to the plus strand are above the horizontal axis in teal and reads mapping to the minus strand are below the horizontal axis in tan. Numbers on *y*-axes indicate read depth. For gene schematic, numbered genes indicate the locus tag for uncharacterized genes (ref-seq annotations begin with the prefix *ABUW_RS*). (*C*) and (*D*) Plating analysis for ∆*lrp ∆csrA* (*C*) and WT (*D*) harboring the indicated *dad* locus expression plasmids on LB (*Top* row) and M9-succinate (*Bottom* row) with increasing concentrations of IPTG. pEV, empty vector, pDadA-X, *dadA dadX* coexpression plasmid. (*E*) Plating analysis for the indicated strains on LB (*Left*) and M9-succinate (*Right*). Plating assays in panels *C*–*E* were conducted as described in the legend for Fig. 1. Numbers to the right indicate the dilution plated.

Lrp family proteins can function as global regulators of hundreds of genes, or as local regulators that control only a few genes (21, 24). Considering this, and because ectopic expression of Lrp had varying effects in the different ∆*csrA* suppressor clones (Fig. 2*C*), we wished to determine the regulatory targets of Lrp in *A. baumannii* AB5075. We performed transcriptome analysis on exponentially growing cells of WT AB5075 and the ∆*lrp* strain using RNA sequencing (RNA-seq) which revealed that the transcript abundance for 748 genes was significantly altered in cells of the *lrp* mutant compared to that of WT cells (Dataset S2). Of the differentially regulated genes, defined as a log_2_ fold-change greater than or less than 1 with a q-value under 0.05, 328 genes had decreased expression in the absence of *lrp,* while 420 exhibited increased expression (Fig. 3*A*). That nearly 20% of the ≈4,000 genes in the AB5075 genome are differentially expressed in the ∆*lrp* strain is consistent with Lrp functioning as a global regulator in *A. baumannii*.

### An Lrp-Controlled Operon Is Responsible for Suppression of ∆*csrA*.

In the ∆*lrp* transcriptome, we noticed strong downregulation of an apparent four gene operon adjacent to the *lrp* locus (Fig. 3*B*). The predicted functions of these four genes relate to D-amino acids, including a D-amino acid dehydrogenase (*ABUW_RS18450*), an alanine racemase (*ABUW_RS18445*), a RidA-family enamine/imine deaminase (*ABUW_RS18440*), and a D-amino acid transporter (*ABUW_RS18435*). Based on homologs in other organisms, we refer here to the dehydrogenase as *dadA*, the racemase as *dadX*, and the transporter as *cycA2*. Interestingly, in three of the four ∆*csrA* suppressors harboring deletions in the *lrp* region, the open reading frames for *dadA, dadX, ABUW_RS18440* (the *ridA* homolog), and *cycA2*–hereafter called the *dad* locus–was also lost (Fig. 2*A*).

The significant decrease in expression of the *dad* locus in ∆*lrp* cells, along with the observation that ectopic expression of *lrp* failed to restore the ∆*csrA* phenotype in CS01, CS02, and CS12–the three suppressor strains that lost the *dad* locus (Fig. 2)–led us to hypothesize that dysregulation of the *dad* operon in the ∆*lrp* background may contribute to suppressing the *csrA* growth phenotype. To test this, we introduced plasmids containing the *dad* locus genes under the control of an IPTG-inducible promoter to WT AB5075 and the ∆*lrp ∆csrA* mutant. In the ∆*lrp ∆csrA* mutant, ectopic expression of *dadA, dadX,* or *cycA2* impaired growth on LB as the concentration of inducer increased, suggesting that these genes may contribute to the ∆*csrA* phenotype (Fig. 3*C*). A plasmid-borne copy of the *ridA* homolog (*ABUW_RS18440*) did not impact growth of ∆*lrp ∆csrA*. Intriguingly, at the highest level of induction (2 mM IPTG), ∆*lrp ∆csrA* cells carrying plasmid-borne copies of *dadA, dadX*, or *cycA2* also displayed reduced growth on M9-succinate. Similarly, in WT cells, ectopic expression of *dadA, dadX,* or *cycA2* led to reduced growth on both M9-succinate and LB as the IPTG concentration increased (Fig. 3*D*). These analyses indicate that increasing *dadA, dadX,* or *cycA2* expression impairs growth of *A. baumannii,* independently of the growth medium and we infer that dysregulation of the *dad* genes interferes with critical aspects of *A. baumannii* physiology and hypothesize that CsrA may negatively regulate one or more genes encoded by the *dad* locus.

We next asked whether the loss of genes in the *dad* operon was sufficient to suppress the ∆*csrA* mutation. In wild-type cells, deletion of individual *dad* genes did not affect growth in LB (Fig. 3*E*). Likewise, ∆*csrA* cells lacking *ABUW_RS18440* failed to grow on LB, suggesting again that the *ridA* homolog does not contribute to the ∆*csrA* phenotype. While strains harboring deletion of either *dadA* or *dadX* in combination with the ∆*csrA* mutation failed to grow in LB, deletion of both *dadA* and *dadX* in the ∆*csrA* strain (i.e., *∆dadA ∆dadX ∆csrA*) did grow in LB, albeit poorly (Fig. 3*E*). Despite multiple attempts, we were unable to recover a ∆*cycA2 ∆csrA* double mutant. Immediately downstream of *cycA2* is *cycA1*, another putative D-amino acid transporter that shares high similarity with *cycA2* (82% nucleotide identity; 88% amino acid identity). We reasoned that *cycA2* and *cycA1* may be functionally redundant and thus attempted to delete *csrA* from a strain lacking both *cycA2* and *cycA1*. We successfully isolated a ∆*cycA1 ∆cycA2 ∆csrA* triple mutant, and like the ∆*dadA ∆dadX* ∆*csrA* strain, the ∆*cycA1 ∆cycA2 ∆csrA* mutant exhibited scant growth on LB (Fig. 3*E*). Together, these results indicate that several *dad* locus genes contribute to the ∆*csrA* growth phenotype and further suggest that D-amino acid homeostasis–possibly alanine specifically–may be altered in cells lacking CsrA. In line with this, the ∆*csrA* mutant exhibited a growth defect when cultured in M9-succinate medium supplemented with L-alanine or D-alanine; in both cases, the loss of *lrp* in the ∆*csrA* background improved growth (Fig. 2*E*).

### CsrA Regulates *dadA* and *cycA2* Translation.

**Fig. 4.**
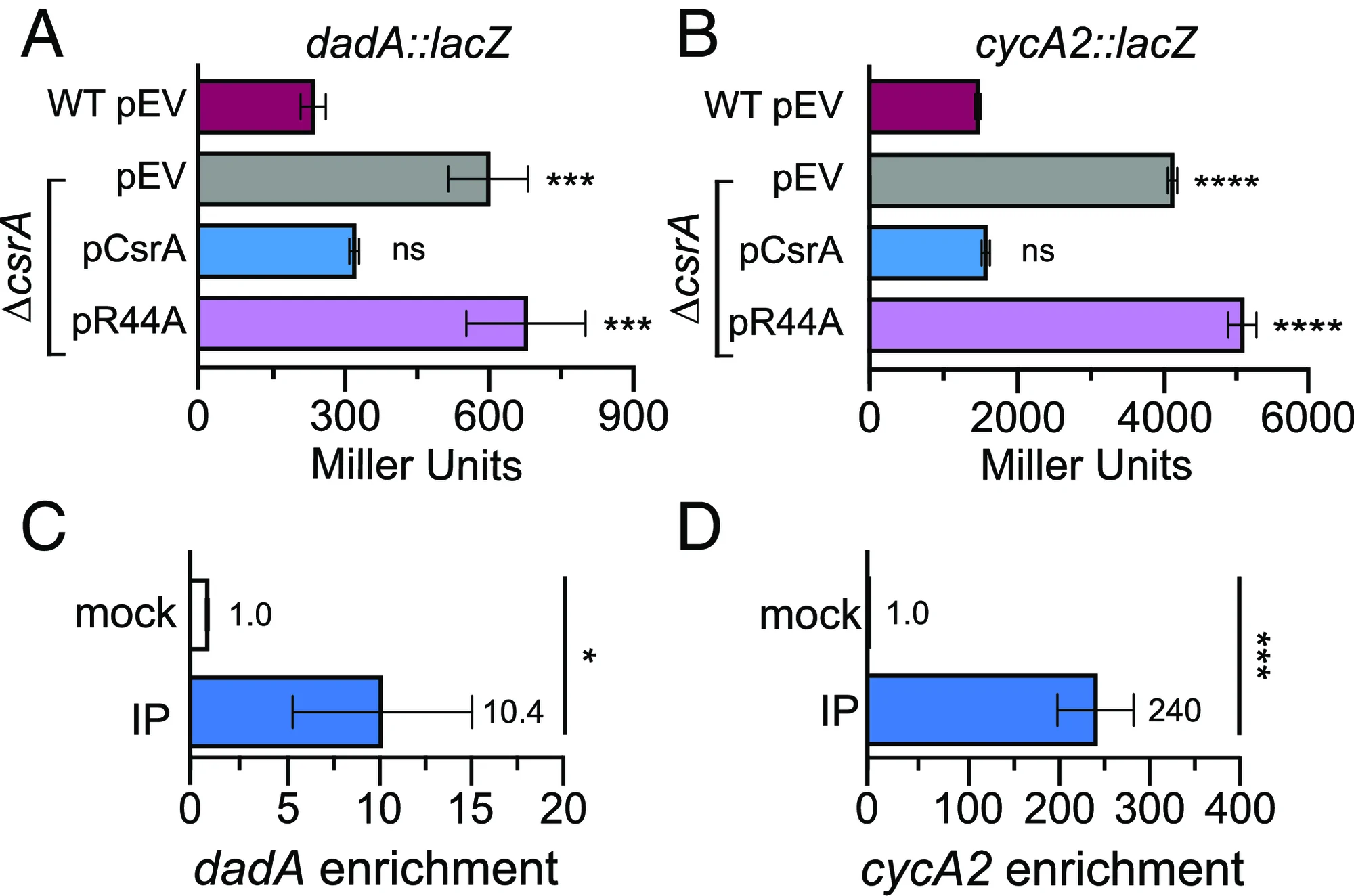
*dadA* and *cycA2* are CsrA targets. Beta-galactosidase assay results from (*A*) *dadA::lacZ* and (*B*) *cycA2::lacZ* translational reporter in wild-type AB5075 or ∆*csrA* harboring empty vector (pEV), wild-type *csrA* (pCsrA), or RNA-binding deficient *csrA* (pR44A) plasmids. (*C* and *D*) RIP-qPCR analysis with CsrA-V showing normalized enrichment for (*C*) *dadA* and (*D*) *cycA2* transcripts. Cultures (n = 3) grown in LB to exponential phase prior to IP; mock, IP performed with no antibody; IP, IP performed with anti-VSV-G antibodies. β-galactosidase assays conducted with exponentially growing cells cultured in M9-succinate containing 20 mM L-alanine and were independently repeated at least twice with biological triplicates; data from a representative experiment shown as the mean activity (in Miller Units); error bars represent one SD. Significance assessed by one-way ANOVA with Dunnett’s post hoc test to correct for multiple comparisons (*A* and *B*) or two-tailed *t* test (*C* and *D*). Asterisks indicate significant differences from WT with pEV for β-galactosidase assays and from mock control for RIP-qPCR; *P < 0.05; ***P < 0.001; ****P < 0.0001; ns, not significant.

The data presented above suggest that transcripts derived from the *dad* locus may be regulatory targets of CsrA and may be dysregulated when *csrA* is deleted. To explore this possibility, we created transcriptional and translational *lacZ* fusions. The reporters included approximately 500 nucleotides upstream of the *dadA* start codon to capture the *dadA* promoter; the transcriptional reporter (P*_dadA_::lacZ)* ends at the predicted *dadA* transcription start and the translational reporter (*dadA::lacZ*) includes the first several codons of the DadA protein. The two reporters were introduced into WT AB5075 and the *∆csrA* strain and assayed for β-galactosidase activity following growth in M9-succinate containing 20 mM L-alanine, which induces *dad*A promoter activity in an *lrp*-dependent manner (*SI Appendix,* Fig. S2). Consistent with *dadA* being a regulatory target of CsrA, the activity of the *dadA::lacZ* translational reporter was significantly higher in the *∆csrA* strain compared to wild-type and this increase could be complemented when a *csrA* plasmid (pCsrA) was introduced (Fig. 4*A*). On the contrary, complementation with an RNA-binding deficient allele of *csrA* (Arginine 44 changed to Alanine, pR44A; 25) did not alter reporter activity, indicating that CsrA requires the ability to bind RNA to regulate *dadA* expression (Fig. 4*A*). Importantly, the P*_dadA_::lacZ* transcriptional reporter did not exhibit increased activity in the ∆*csrA* background (*SI Appendix,* Fig. S2), indicating that the increased signal observed with the *dadA::lacZ* translational reporter is not due to increased *dadA* promoter activity in the cells lacking *csrA*. Analogous experiments conducted with *cycA2::lacZ* translational (Fig. 4*B*) and P*_cycA2_*::lacZ transcriptional (*SI Appendix,* Fig. S3) reporters produced similar results, indicating that both *dadA* and *cycA2* are translationally repressed by CsrA. To extend these analyses, we introduced the *cycA2::lacZ* translational reporter plasmid into additional *A. baumannii* isolates, including strains AB0057, AYE, ACICU, and ATCC 17978, and measured reporter activity for cells with an empty vector or pCsrA. In each strain background, the reporter activity decreased in the presence of the CsrA plasmid, indicating that CsrA’s control over *cycA2* is most likely conserved across the *A. baumannii* species (*SI Appendix,* Fig. S2).

CsrA family proteins recognize specific RNA motifs in their RNA targets, including RUACARGGAUGU in *E. coli* (26) and CANGGAYG in *Pseudomonas aeruginosa* (27), with the GGA nucleotides comprising a core recognition element for CsrA binding. Near the translation initiation region for both *dadA* and *cycA2*, there are two potential CsrA-binding motifs (referred to hereafter as GGA motifs) positioned upstream of the *dadA* and *cycA2* start codons (*SI Appendix,* Fig. S3). If CsrA exerts its regulatory effects by associating with mRNAs encoding *dadA* and *cycA2*, we reasoned that mutation of the GGA motifs would abolish CsrA mediated repression of the translational reporters. Indeed, the activity of the mutant reporters where both GGA motifs were changed to AAG (*dadA^AAG^::lacZ* and *cycA2^AAG^::lacZ*) no longer responded to CsrA (*SI Appendix,* Fig. S3). Collectively, the results of the *lacZ* reporter assays are consistent with CsrA repressing the translation of mRNAs encoding *dadA* and *cycA2* by associating with GGA motifs located near the start codons of each open reading frame.

We next asked if CsrA interacts with mRNAs encoding *dadA* and *cycA2*, using RNA-immunoprecipitation and reverse-transcriptase qPCR (RIP-qPCR) to do so. We first introduced a Vesicular Stomatitis Virus-G protein (VSV-G) epitope to the C-terminus of the native *csrA* (CsrA-V). A similar strategy was previously used to study RsmA, a CsrA homolog in *P. aeruginosa* (28), and, importantly, we did not observe any growth differences between WT AB5075 and the isogenic CsrA-V strain (*SI Appendix,* Fig. S4). RIP-qPCR with the CsrA-V strain allowed us to detect robust enrichment of CsrB, a regulatory RNA known to interact with CsrA (*SI Appendix,* Fig. S3) (20). We also detected enrichment of *dadA* and *cycA2*, indicating that CsrA can directly interact with transcripts encoded by the *dad* locus (Fig. 4 *C* and *D*). When we examined the *lrp* transcript by RIP-qPCR, we failed to detect enrichment, suggesting that *lrp* is not a direct CsrA target in *A. baumannii* (*SI Appendix,* Fig. S3). Consistent with this observation, the activity of an *lrp::lacZ* translational fusion reporter strain was unchanged in ∆*csrA* cells compared to wild type AB5075 although there was increased activity for a transcriptional *lrp::lacZ* reporter in the ∆*csrA* background (*SI Appendix,* Fig. S2). Collectively, the RIP-qPCR analyses and the *lacZ* reporter data presented above support a model where CsrA posttranscriptionally regulates the translation of the transcript(s) encoding *dadA* and *cycA2*.

### DadA Activity Is Increased in the ∆*csrA* Background.

**Fig. 5.**
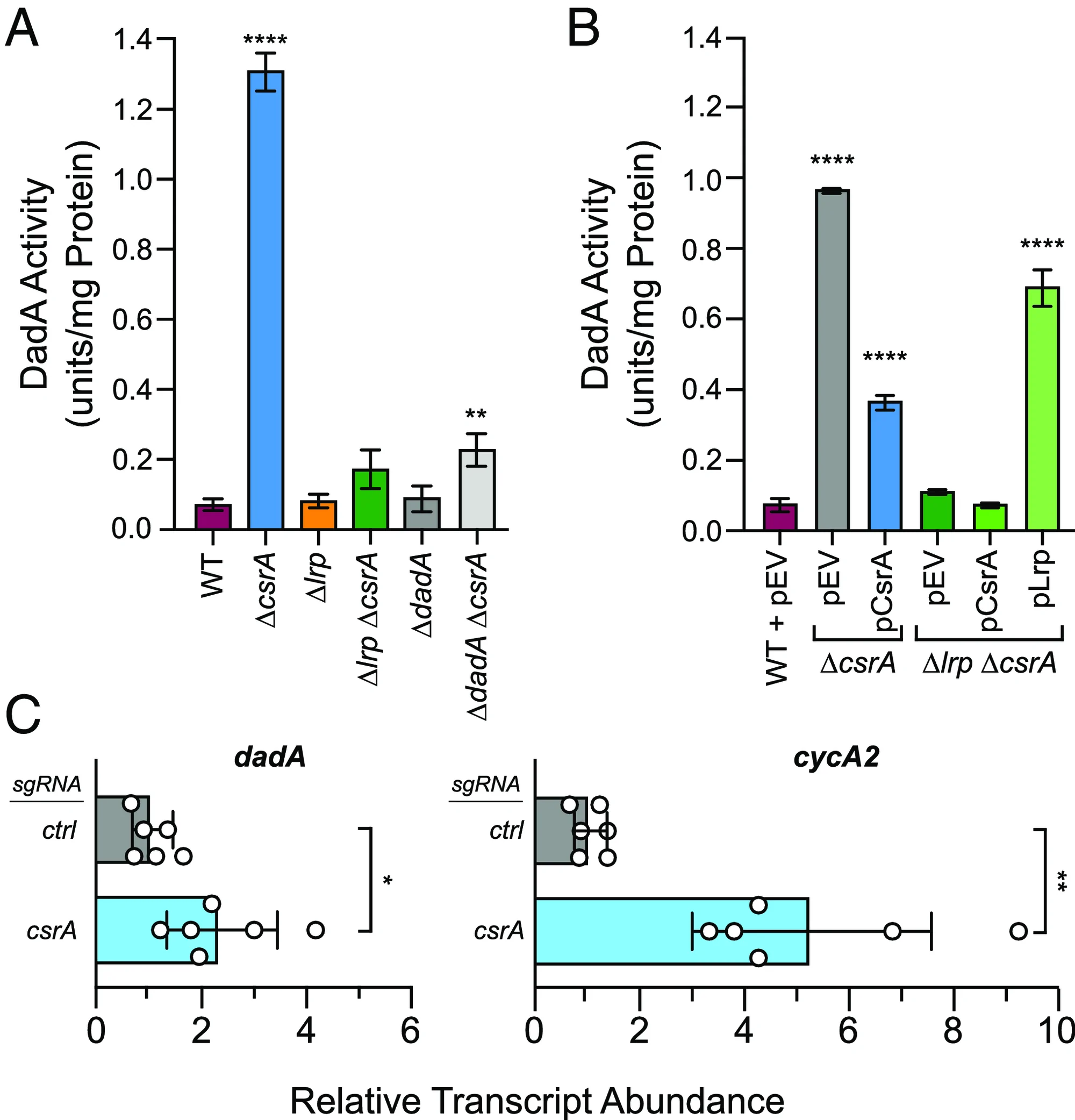
CsrA negatively regulates DadA and CycA2. (*A* and *B*) DadA enzymatic activity assay. DadA activity, reported as units/mg total protein, was measured in lysates prepared from cells cultured overnight in M9-succinate medium supplemented with 20 mM L-alanine for the indicated strains (*A*) and strains harboring the indicated plasmids (*B*). Data depicted as the mean DadA activity from biological triplicate cultures; error bars represent one SD from the mean. (*C*) Transcript abundance for *dadA* and *cycA2* following depletion of *csrA* via CRISPRi. RNA collected from AB5075 attTn*7*::dCas9 expressing a control sgRNA (i.e., nontargeting; *ctrl*) or *csrA*-targeting sgRNA (*csrA*) at exponential phase assessed by quantitative RT-PCR. Fold-change determined relative to *recA* transcript using the comparative Ct method (2^–∆∆CT^); experiment was repeated twice and data from both experiment runs are shown as the mean relative fold change (bars) and individual data points (circles); error bars represent one SD of the mean. Statistics assessed via one-way ANOVA with Dunnett’s post hoc test to correct for multiple comparisons (*A* and *B*) or via two-tailed *t* test (*C*). Asterisks indicate significant differences from WT (*A*), WT with pEV (*B*), or WT expressing a nontargeting sgRNA (*C*); *P < 0.01; **P < 0.005; ****, *P <* 0.0001; ns, not significant.

Annotated as a D-amino acid dehydrogenase, DadA is predicted to convert D-alanine to pyruvate. Because DadA expression is repressed by CsrA (Fig. 4*A*), and the ectopic expression of *dadA* is toxic (Fig. 3 *C* and *D*), we wondered whether cells lacking *csrA* exhibit increased DadA enzymatic activity. To test this, we conducted a D-amino acid dehydrogenase assay (hereafter referred to as the Dad assay) that measures the conversion of D-alanine to pyruvate in cell lysates (29). Consistent with our prior observations, cell lysates from the ∆*csrA* strain exhibit increased DadA activity compared to WT (Fig. 5*A*). Importantly, the increased Dad activity observed in the ∆*csrA* mutant is largely attributable to DadA, as the activity measured in the ∆*dadA ∆csrA* strain is significantly reduced relative to the ∆*csrA* strain. Complementation of the ∆*csrA* strain with pCsrA reduced DadA activity, whereas introducing pCsrA to the *∆lrp ∆csrA* mutant did not alter DadA activity compared to the empty vector (Fig. 5*B*). In this same background, however, expression of *lrp* from a plasmid causes a significant increase in DadA activity, to a level on par with the ∆*csrA* strain (Fig. 5*B*). Collectively, these findings are consistent with our previous data and support a model where Lrp activates the transcription of *dadA* and CsrA represses *dadA* translation.

### CsrA Influences the Transcript Abundance of *dadA* and *cycA2*.

One limitation of the experiments detailed above is that they were largely performed in conditions where CsrA is not essential (i.e., in M9 minimal media) and thus may not fully recapitulate what happens to cells growing in conditions where CsrA is essential for growth (i.e., rich media). To more directly determine the role of CsrA under CsrA-required conditions, we utilized CRISPRi to deplete CsrA during growth in LB. Importantly, when we targeted *csrA* with CRISPRi, growth in rich media was impaired at exponential phase and CsrA-V protein abundance was decreased (*SI Appendix*, Fig. S4). We subsequently collected RNA from exponentially growing cultures (OD_600_ = 0.3 to 0.5) of the CsrA-V-*dCas9* strain harboring either a control sgRNA plasmid or the *csrA* sgRNA plasmid. As shown in Fig. 5*C*, cells expressing the *csrA* sgRNA had increased transcript abundance of both *dadA* and *cycA2*, by 2.4- and 5.3-fold, respectively. Thus, in rich media, CsrA negatively impacts the transcript abundance for both *dadA* and *cycA2*. These data also suggest that loss of CsrA results in increased *dad* gene expression, which likely contributes to the growth defects associated with loss of *csrA*.

### Cells Lacking *csrA* Exhibit Envelope Integrity Defects.

**Fig. 6.**
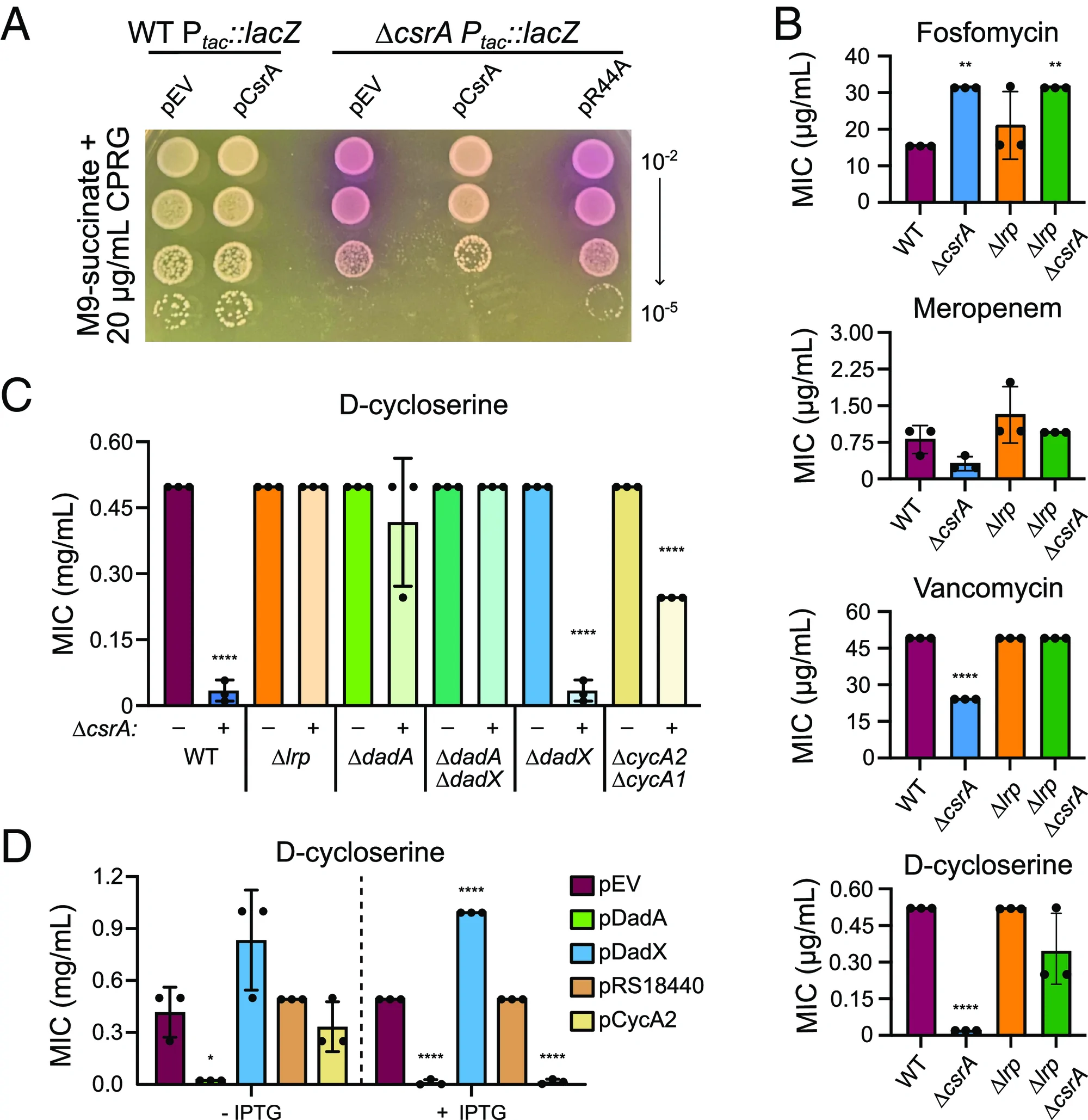
Cells lacking *csrA* have impaired cell envelope integrity. (*A*) Dilutions of AB5075 (WT) and ∆*csrA* with *P_tac_::lacZ* reporter harboring empty vector (pEV), wild-type *csrA* (pCsrA), or RNA-binding deficient *csrA* (pR44A) plasmids on M9-succinate agar with 20 µg/mL chlorophenyl red-β-D-galactopyranoside (CPRG); pink pigment indicates CPRG cleavage. Numbers to the right indicate the dilution plated. (*B*–*D*) Minimum inhibitory concentration (MIC) determination for the indicated antibiotics and strains (*B* and *C*) or in WT cells harboring the indicated plasmid (*D*). MIC assays were conducted in biological triplicate in M9-succinate medium. Experiments were independently repeated three times; results from a representative run are shown. Dots in panels *B*–*D* represent MIC values for the three replicates included in the experiment. Statistics assessed via one-way ANOVA with Bonferroni post hoc correction for multiple comparisons. Asterisks indicate significant differences compared to wild-type cells (panels *B* and *C*) or empty vector control (pEV; panel *D*); *P < 0.05; **P < 0.01; ****P < 0.0001; comparisons not reaching significance are not indicated.

Alanine is a critical component of the bacterial cell wall, as the pentapeptide side chains that comprise PG monomers begin with an L-alanine residue and end with two sequential D-alanine residues (reviewed in ref. 30). We thus wondered whether the observed dysregulation of the *dad* locus in the *∆csrA* background might impact cell envelope integrity in *A. baumannii*. Indeed, previous reports observed that ∆*csrA* mutants display morphological defects including elongated cells, which allude to possible cell wall or cell division defects (13). To investigate whether the ∆*csrA* mutant has an impaired envelope, we grew cells on M9-succinate plates containing chlorophenyl red-β-D-galactopyranoside (CPRG), a β-galactosidase substrate that is unable to cross the envelope of gram-negative bacteria (31). Therefore, *lacZ*-positive bacterial cells will only cleave CRPG if the strain exhibits envelope defects (i.e., an impaired cell wall or increased cellular lysis); CPRG cleavage is detected visibly as the cleaved product, chlorophenyl red (CPR), is red. To assess the integrity of the ∆*csrA* envelope, we used a derivative of strain AB5075 harboring the *E. coli* *lacZ* gene at the Tn*7* attachment site (WT P*_tac_::lacZ*) (22) and deleted *csrA* from that strain (∆*csrA* P*_tac_*::*lacZ).* Importantly*, lacZ* reporter activity was equivalent in both of these strains (*SI Appendix,* Fig. S5). When plated on CPRG-containing M9-succinate plates, the ∆*csrA* P*_tac_*::*lacZ* strain generated robust CPRG cleavage, consistent with the ∆*csrA* strain having impaired envelope integrity, while the wild-type strain did not generate any red pigment (Fig. 6*A*). In fact, we only detected CPR production surrounding wild-type cells in the presence of the cell wall targeting antimicrobial meropenem, indicating that CPRG is normally unable to cross the *A. baumannii* membrane, as anticipated (*SI Appendix,* Fig. S5). Complementation of the ∆*csrA* strain with wild-type pCsrA, but not the RNA-binding deficient R44A allele, decreased CPR production, indicating that the increased CPR production in the ∆*csrA* strain results from the loss of CsrA’s role in posttranscriptional regulation (Fig. 6*A*). We thus conclude that the ∆*csrA* mutant has cell envelope defects that result in increased cellular permeability and/or an increased frequency of cell lysis.

We next investigated if the ∆*csrA* mutant also exhibits altered sensitivity to cell wall targeting antibiotics and determined the minimum inhibitory concentration (MIC) for four different cell wall-active antibiotics: D-cycloserine, fosfomycin, meropenem, and vancomycin. These antibiotics target discrete steps within the PG biosynthetic pathway: D-cycloserine is a structural analog of D-alanine that inhibits alanine racemase and D-alanine ligase (29); fosfomycin irreversibly inhibits MurA which is involved in generating the lipid II PG carrier (reviewed in ref. 32); meropenem inhibits penicillin-binding proteins; and vancomycin binds to the terminal D-ala – D-ala dipeptide on PG monomers and prevents PG cross-linking. Compared to wild-type, the ∆*csrA* mutant was more sensitive to meropenem, vancomycin, and D-cycloserine (Fig. 6*B*). MIC values for the ∆*lrp* mutant were not significantly different than wild-type for any of the four antimicrobials tested. Interestingly, the ∆*lrp ∆csrA* double mutant had an intermediate MIC value for D-cycloserine, being more sensitive than wild-type and ∆*lrp*, but more resistant than the ∆*csrA* strain. Both ∆*csrA* and *∆lrp ∆csrA* displayed increased resistance to fosfomycin compared to wild-type cells (Fig. 6*B*).

To ascertain whether the altered sensitivity to cell-wall targeting antibiotics in the ∆*csrA* background results from the dysregulation of the *dad* locus, we performed two follow-up experiments. We first determined MICs in strains lacking individual *dad* genes in both wild-type and ∆*csrA* backgrounds. For vancomycin and fosfomycin, no significant differences were observed in *dad* locus mutants with respect to the *csrA* deletion, indicating that the *csrA-*mediated changes in drug resistance for these antimicrobials are independent of the *dad* locus (*SI Appendix,* Fig. S6). Meropenem resistance in the *∆dadA ∆csrA* strain and the ∆*cycA2 ∆cycA1 ∆csrA* triple mutant strains increased relative to the ∆*csrA* strain, but the observed differences were less than twofold, making it difficult to ascribe a definitive role for DadA or CycA2 in the meropenem resistance phenotype associated with the *∆csrA* mutation (*SI Appendix,* Fig. S6). The increased sensitivity of the ∆*csrA* strain for D-cycloserine, however, does appear to result from dysregulation of *dadA* and/or *cycA2*, as both the ∆*dadA ∆csrA* and *∆cycA1 ∆cycA2 ∆csrA* strains exhibit an MIC closer to wild type cells than that of the *∆csrA* mutant (Fig. 6*C*). The loss of *csrA* in the ∆*dadX* background mirrored ∆*csrA*, indicating that *dadX* does not contribute to D-cycloserine resistance in the *∆csrA* background (Fig. 6*C*). We next examined D-cycloserine resistance during ectopic expression of the *dad* genes in wild-type AB5075. This scenario should mimic the *csrA* deletion background, where the *dad* genes are aberrantly over expressed (Figs. 4 and 5). In these analyses, ectopic expression with 20 µM IPTG (which does not cause toxicity, see Fig. 3) of either *dadA* or *cycA2* significantly reduced D-cycloserine resistance, whereas ectopic expression of *dadX* led to a slight increase in D-cycloserine resistance in wild-type AB5075 (Fig. 6*D*). Because D-cycloserine inhibits alanine racemase enzymes like DadX, we speculate that the moderate increase in DadX abundance produced under the low-induction conditions used here may provide some protection. Consistent with other experimental data, ectopic expression of the *ridA* homolog encoded by *ABUW_RS18440* did not impact D-cycloserine resistance (Fig. 6*D*). Collectively, these analyses indicate that the increased expression of *dadA* and *cycA2* are important drivers of the increased D-cycloserine sensitivity exhibited by cells lacking *csrA*.

## Discussion

**Fig. 7.**
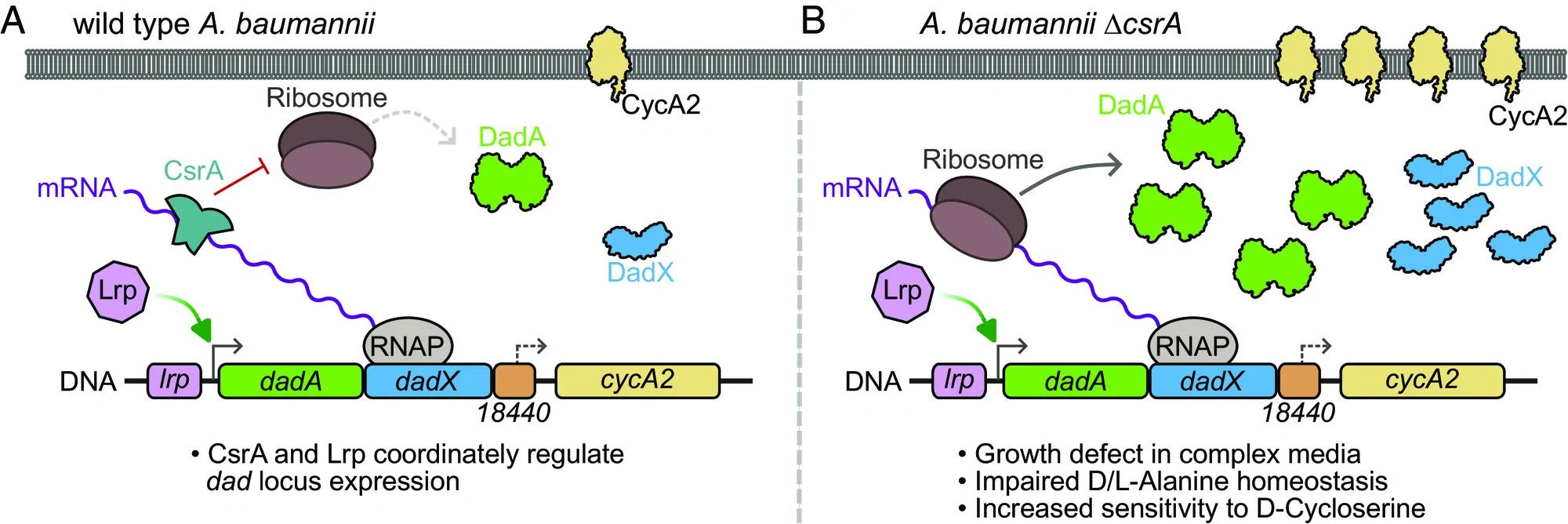
Model for regulation of the *dad* locus by CsrA and Lrp. (*A*) In wild-type cells, Lrp activates transcription of the *dad* locus in response to alanine. CsrA represses translation of *dadA* and *cycA2* to maintain homeostatic expression. (*B*) In cells lacking *csrA*, Lrp still activates *dad* locus transcription. The resulting increased translation of the *dad* locus gene products results in accumulation of DadA, DadX, and/or CycA2, which leads to dysregulated alanine homeostasis, impaired cell envelope integrity and sensitivity to cell-wall targeting antibiotics like D-cycloserine.

Posttranscriptional regulation plays key roles in governing gene expression across the bacterial domain. This is especially true for *A. baumannii*, where the highly conserved posttranscriptional regulators, Hfq (essential) and CsrA (conditionally essential), are critical for maintaining cellular physiology (13–15, 22, 23, 33). We leveraged the conditional essentiality of CsrA through a suppressor search to identify mutations that allow a ∆*csrA* strain to grow in rich media. These efforts led to the isolation of 12 *csrA* suppressor clones, several of which contained large deletions in the *dad* locus. This finding suggests that the dysregulation of one or more genes in this region may drive the ∆*csrA* growth phenotype. Our follow-up analyses revealed that CsrA represses the translation of multiple transcripts encoded by the *dad* locus, and the resulting overexpression of these genes contributes to the growth defects associated with loss of *csrA* (see model in Fig. 7). We propose that the resulting dysregulation of D-alanine metabolic processes in the absence of *csrA* significantly impacts *A. baumannii* cell envelope integrity, as the ∆*csrA* strain exhibits increased cellular permeability and altered sensitivity to several cell-wall targeting antibiotics.

Central to the Dad/Lrp region is the Lrp transcription factor. In other bacteria, Lrp family regulators sense the nutritional environment through small molecule ligands and regulate expression of diverse genes involved in pathways ranging from metabolism and virulence to motility and antimicrobial resistance (21). The ligands recognized by Lrp family proteins thus far are largely amino acids, with direct evidence for several amino acid-Lrp pairs. *E. coli* Lrp binds leucine and alanine, with either amino acid driving similar regulatory outcomes (34, 35). The *Mycobacterium tuberculosis* regulator LrpA recognizes aromatic amino acids with an apparent preference for phenylalanine and histidine (36). Crystallographic studies in *Neisseria meningitidis* indicated that its Lrp homolog can bind leucine and methionine (37). In the case of *A. baumannii,* our experiments indicate that alanine may serve as a ligand for Lrp, as the activity of a P*_dadA_*::lacZ transcriptional reporter is activated by alanine in an *lrp*-dependent fashion (*SI Appendix,* Fig. S2). It will be important to establish whether additional amino acids influence the activity of the *A. baumannii* Lrp in future studies.

Lrp family proteins act either as global regulators, controlling expression of hundreds of genes or as local regulators that control only a few (21). For *A. baumannii*, our transcriptomic analyses indicate that Lrp functions as a global regulator, with a regulon approaching 750 genes (Fig. 3 and Dataset S2). Not surprisingly, the predicted functions of the Lrp regulon span many categories, including metabolism and motility (i.e., components of the type IV pilus). Most pertinent to CsrA, however, is our finding that Lrp activates the expression of the *dad* locus, including *dadA, dadX, ABUW_RS18440* (a predicted RidA family protein), and *cycA2.* This locus initially caught our interest because these genes are missing in three of the four DadA/Lrp region suppressors, and Lrp is positioned immediately upstream and transcribed divergently from *dadA* in *A. baumannii* (Fig. 3). In *E. coli*, *dadA* and *dadX* comprise an operon, while *cycA* is transcribed from a different genomic location, but despite the different genetic context, the *E. coli* Lrp directly regulates transcription of the *dadAX* operon and *cycA* (38–40). Recent work from the Freddolino group indicates that Lrp influences the expression of nearly one third of the *E. coli* genome through direct and indirect mechanisms (39). We speculate that the *A. baumannii* Lrp protein functions similarly, as among the Lrp-regulated genes, 36 encode predicted DNA-binding transcription factors. It will thus be of interest to determine the direct regulatory targets for Lrp in *A. baumannii.*

Our data indicate that CsrA negatively regulates expression of the *dad* locus, likely through direct interactions. We also considered the possibility that the *lrp* transcript may be a regulatory target of CsrA in *A. baumannii*, which was previously observed in *E. coli* (41). Similarly, in *Vibrio cholerae*, transcriptomic data indicate that cells expressing an RNA-binding deficient CsrA allele exhibit increased expression of *lrp* transcripts at multiple growth stages, although whether this reflects CsrA directly targeting the *lrp* mRNA transcript was not determined (42). We did not detect enrichment of *lrp* transcripts RIP-qPCR with CsrA-V (*SI Appendix,* Fig. S3), nor did we detect differences in activity of an *lrp::lacZ* translational fusion reporter between wild-type and ∆*csrA* (*SI Appendix,* Fig. S2). We do note, however, that the loss of *csrA* may indirectly impact *lrp* expression, as a P*_lrp_::lacZ* transcriptional showed increased activity in the ∆*csrA* background, although this did not complement when we introduced a plasmid-borne copy of *csrA* (*SI Appendix,* Fig. S2). Thus, while CsrA may indirectly regulate *lrp*, the translational reporter data and the RIP-qPCR data argue against CsrA directly associating with the *lrp* mRNA.

One important question that remains unanswered is how CsrA and Lrp, both of which are global regulators in *A. baumannii*, coordinate the expression of not only the *dad* locus but also their other regulatory targets. For the *dad* locus, our evidence indicates that Lrp activates transcription in response to alanine (*SI Appendix,* Fig. S2) and that the *dad* transcript(s) are repressed by CsrA (Fig. 4). What mechanisms might be in place to counteract CsrA-mediated repression of the *dad* transcript when alanine is present? One possibility is that CsrA levels are stable across different growth conditions and CsrA essentially buffers against overexpression of the *dad* transcripts. Alternatively, Lrp may indirectly control CsrA activity by modulating the expression of one or more regulatory RNAs that sequester CsrA, such as CsrB (20). Intriguingly, CsrB transcript abundance increased approximately twofold in the *∆lrp* strain background (Dataset S2), suggesting that a feedback mechanism to coordinate the activities of Lrp and CsrA may exist. Given the importance of finely tuning expression of the *dad* locus, it is imperative to determine how these two global regulators coordinate their respective activities.

Here we sought to uncover the genetic basis for the conditional essentiality of *csrA* in *A. baumannii.* Through a suppressor search, we determined that loss of *csrA* results in dysregulation of a suite of genes involved with D-alanine metabolism. These genes, whose transcripts are translationally repressed by CsrA, are toxic when ectopically expressed, even in wild type *A. baumannii*. We suspect that this dysregulation manifests as impaired cellular envelope integrity in the *∆csrA* mutant and further alters the antibiotic resistance profile, particularly for cell-wall targeting antimicrobials. Taken together, our findings reveal an intricate balancing act between CsrA and Lrp, two global regulators that coordinate the expression of several genes involved with D-amino acid metabolism. CsrA dampens the expression of potentially toxic proteins, implying that CsrA is essential because it counters the activity of Lrp, which drives the expression of the *dad* locus. Our data thus provide additional complexity regarding how bacteria utilize posttranscriptional regulation to protect against potentially toxic effects of transcription.

## Materials and Methods

### Bacterial Strains and Plasmids.

Bacterial strains, plasmids, and oligonucleotides are listed in *SI Appendix*, Tables S1–S3*. A. baumannii* AB5075 and mutant strains thereof were cultured in M9 media supplemented with 20 mM of carbon source(s), such as succinate and alanine or in LB. *A. baumannii* strain AB5075 is known to switch between opaque and translucent subpopulations and thus experiments conducted herein potentially contained a mixture of cells in these two phases (20, 43). All plasmids and mutant strains were confirmed by sequencing and/or PCR prior to use. Specific details for plasmid and strain construction can be found in *SI Appendix*.

### Isolation of ∆*csrA* Suppressors.

Individual colonies of ∆*csrA* were inoculated into 5 mL of LB and incubated at 37 °C with shaking (200 rpm). Every 24 h, cultures were diluted 1:100 into a fresh portion of LB media until cultures were turbid, at which point 100 µL was removed and plated onto solid LB. Suppressors typically arose in the second LB culture. Isolated colonies of the candidate suppressors were purified to M9-succinate plates and growth on LB was confirmed the following day. Colonies that retained growth on LB following passage through M9-succinate were stored at −80 °C in a 20% glycerol solution for further analysis. A total of 12 independent suppressors were isolated from two independently derived ∆*csrA* strains (six clones from each ∆*csrA* parent).

### Whole Genome Sequencing of Suppressors.

Bacterial genomic DNA was isolated using the MasterPure DNA Purification Kit (Biosearch Technologies) following the manufacturer’s recommendations. Whole genome sequencing (WGS) was performed by SeqCenter (Pittsburg, PA). WGS analysis was performed with Geneious Prime (v 11.0.20.1+1) and mutations identified are listed in Table 1 and Dataset S1. Raw WGS sequencing reads have been deposited in the NCBI Sequence Read Archive (SRA) under BioProject ID: PRJNA1391534 (44).

### β-Galactosidase Assays.

β-galactosidase assays were conducted essentially as described previously (45). Briefly, triplicate overnight cultures of the indicated reporter strains were diluted 1:50 in fresh media and cultured until early exponential phase (OD_600_ ≈ 0.1 to 0.2). Bacterial cells were permeabilized with chloroform and SDS and β-galactosidase activity was determined using 2-nitro-phenyl-β-D-galactopyranoside (ONPG). Assays were independently repeated at least three times; average values from one representative run are reported. Statistical analyses were conducted by one-way ANOVA with Dunnett’s multiple comparison test using GraphPad Prism version 10.4.2.

### D-Amino Acid Dehydrogenase Assay.

DadA activity was assayed using a modified D-amino acid dehydrogenase protocol (29). AB5075 strains and its derivatives were grown in M9-succinate supplemented with 20 mM L-alanine overnight at 37 °C with shaking at 200 rpm. The next day, 1 OD_600_ unit of cells were washed in phosphate buffer, permeabilized with toluene and diluted 1:10 in phosphate buffer containing 20 mM D-alanine and incubated at 37 °C for 30 min after which 250 µL of 2,4-dinitrophenylhydrazine (0.17 mg/mL in 1.2 N HCl) was added to the reaction tubes. The reactions were mixed, incubated for 15 min at room temperature, and neutralized with 1 mL of 2.5 N NaOH for 10 min at room temperature. The OD_520_ of each reaction was recorded and normalized to total protein content as described in *SI Appendix*. Results are reported as DadA units/mg of protein; assays were conducted with biological triplicate cultures and technical duplicates; at least three replicate runs were completed.

### RNA Extraction and RT-qPCR.

RNA was extracted from two mL of culture using the Direct-zol RNA Miniprep kit (Zymo Research, cat. no. R2052) following the manufacturer’s recommendations. After extraction, one µg of RNA was converted to cDNA using LunaScript RT SuperMix (New England Biolabs, cat. no. E3010) as recommended by the manufacturer. Prior to use in qPCR reactions, the cDNA was diluted 1:10. qPCR reactions were performed using iTaq Universal SYBR Green Supermix (BioRad, cat. no. 1725122), and samples were run with technical triplicate reactions. The relative abundances of target genes were normalized to a control transcript (*recA*) using the 2^−∆∆CT^ method as described previously (46). RT-qPCR experiments were independently repeated twice with biological triplicate cultures; results are shown as the mean change in relative transcript abundance for each sgRNA. Error bars represent one SD of the mean. Statistical analysis was conducted with a two-tailed *t* test. Prior to use, all primers used in RT-qPCR were validated for efficiency using a standard curve and melt-temp analysis.

### RNA Immunoprecipitation.

Samples for RNA Immunoprecipitation (RIP) were collected as previously described (46, 47). Briefly, 40 OD_600_ units of exponentially growing AB5075 CsrA-V were processed for RIP as described in *SI Appendix*; parallel samples were processed without adding antibodies as a mock IP control. Following IP, RNA was extracted, converted to cDNA, and subjected to RT-qPCR as described above with the exception that GlycoBlue (Ambion, cat. no. AM9515) was added to aid RNA extraction. The relative abundance of target transcripts was normalized to that of *recA* as a control. Data are reported as the enrichment detected in the IP samples compared to the no antibody control. RIP-qPCR experiments were repeated twice with independent biological triplicates. The mean relative enrichment from all six RIP replicates with error bars representing one SD of the mean are shown.

### RNA-Sequencing.

RNA was extracted from four mL of triplicate cultures of exponentially growing (OD_600_ ≈ 0.5) wild-type AB5075 and the ∆*lrp* strain in LB. DNAse treatment, library preparation, and Illumina sequencing were performed by SeqCenter (Pittsburgh, PA) to produce 150 bp paired end reads. Reads were aligned to the AB5075 genome using Bowtie2 (48) and differential gene expression was determined using HTSeq (49) and DESeq2 (50). A log_2_ fold change value greater than/less than 1 and an adjusted *P*-value of less than 0.05 were used to determine significance. Integrated Genomics Viewer (IGV) software was used for visualization of RNA-seq data (51). For the RNA-seq data, the raw sequence read files and processed files have been deposited with the Gene Expression Omnibus repository under accession number GSE313900 (52).

### CPRG Assay.

Cell wall integrity was assessed using a chlorophenyl red-β-D-galactopyranoside (CPRG) assay (31). Strains containing P*tac*::*lacZ* at the Tn*7* attachment site (22) were serially diluted and spotted onto M9-succinate plates containing apramycin (25 µg/mL) and CPRG (20 µg/mL). Plates were incubated at 37 °C overnight and photographed. CPRG assay was repeated independently on different days with freshly transformed strains for a total three total replicate runs.

### MIC Testing.

MIC testing was performed in a 96-well plate in biological triplicate for each strain. Overnight cultures were refreshed into M9-succinate (1:50) and allowed to grow for approximately 6 h; when necessary, apramycin was added for plasmid maintenance. Strains were then diluted to OD_600_ = 0.002 and added to the wells at a 1:2 dilution (final bacterial concentration OD_600_ = 0.001). Plates were incubated at 37 °C for 24 h. MIC values were determined using the OD_600_ values of the wells compared to control wells containing no antibiotic and no bacteria. MIC testing was independently repeated at least three times. The results from three representative experiments are shown.

## Supplementary Material

Appendix 01 (PDF)

Dataset S01 (XLSX)

Dataset S02 (XLSX)

## Data Availability

Illumina sequencing data have been deposited in the NCBI Gene Expression Omnibus under Series GSE313900 (52) and NCBI BioProject PRJNA1391534 (44). All other data are included in the article and/or supporting information.
