# Supplementary material for: Loss of CsrA in Acinetobacter baumannii leads to lethal dysregulation of alanine metabolism
Source: Proc Natl Acad Sci U S A. 2026 Sep 29;123(40):e2624702123. doi: 10.1073/pnas.2624702123 (PMC13643297; doi:10.1073/pnas.2624702123)
Supplement: Supplementary file 1 [file pnas.2624702123.sapp.pdf]

**Supporting Information for**
Loss of CsrA in *Acinetobacter baumannii* leads to lethal dysregulation of alanine metabolism

Rosa L Sava and Michael J Gebhardt

Michael Gebhardt
Email: Michael-gebhardt@uiowa.edu

**This PDF file includes:**

Supporting text
Figures S1 to S6
Tables S1 to S3
Legends for Dataset S1 to S2
SI References

**Other supporting materials for this manuscript include the following:**

Dataset S1
Dataset S2

**Supporting Information: Methods**

*Growth Conditions*

The base M9 growth medium was comprised of 6 g/L $Na_2PO_4$, 3 g/L $KH_2PO_4$, 0.5 g/L NaCl, 1 g/L $NH_4Cl$, 0.2 mM $CaCl_2$, and 1 mM $MgSO_4$; Lysogeny broth (LB) contained 10 g/L NaCl, 10 g/L tryptone and 5 g/L yeast extract. When needed, agar (15 g/L; Gibco) was added to solidify these media. *Escherichia coli* strain DH5α-F′-*lacI$^q$* (hereafter DH5α) was used for cloning purposes. Antibiotics were added to growth media as needed at the following concentrations: apramycin, 50 μg/mL (solid) and 25 μg/mL (liquid), hygromycin, 250 μg/mL (solid) and 125μg/mL (liquid), tetracycline, 10μg/mL, kanamycin, 50 μg/mL (solid) and 25 μg/mL (liquid), carbenicillin, 100 μg/mL (solid) and 50 μg/mL (liquid), chloramphenicol, 5 μg/mL.

*Plasmid Construction*

Plasmids created for this study were assembled using isothermal assembly (ITA; 1). Plasmid backbones were digested with restriction enzymes (NEB) and purified through gel electrophoresis and spin column clean up. PCR inserts were amplified using KODX polymerase (Millipore Sigma) from wild-type AB5075 genomic DNA using the oligonucleotides indicated in Table S3 and resolved through gel electrophoresis. Products were purified following gel electrophoresis using column clean up. Isothermal Assembly reactions were prepared on ice in a 10 μL volume comprised of 1X ITA mix, digested plasmid backbone, and purified PCR products. Reactions were incubated at 55°C for 20 min and transformed into chemically competent DH5a F'IQ *E. coli* cells. Assembled plasmids were confirmed by Sanger sequencing (Iowa State University DNA Facility) or by whole plasmid sequencing (Plasmidsaurus).

All deletion vectors in *A. baumannii* AB5075-UW were made using the allele exchange plasmid, pMJG42, which carries a tetracycline resistance marker as well as *sacB* to confer sensitivity to sucrose (2). All derivates were constructed using ITA with pMJG42 linearized by digestion with NotI and SpeI. For each deletion, two PCR products were amplified using a gene-specific external forward and internal reverse primer pair (product 1) and an internal forward and external reverse primer pair (product 2) as annotated in Table S3. With each primer set, marker-less deletions were constructed wherein the deletion allele consisted of the native start codon, a threealanine linker, and the native stop codon. As an example, the Δ*lrp* deletion plasmid was created using PCR products derived from primers Ex_Fwd_*lrp* and In_Rev_*lrp* (product 1) and primers In_Fwd_*lrp* and Ex_Rev_*lrp* (PCR product 2). These primers amplify 1 kb upstream of the *lrp* start codon (product 1) and 1 kb downstream (product 2) of the *lrp* stop codon. When combined by ITA, the two products create the 'start codon-alanine linker-stop codon' construct for the *lrp* deletion.

For *lacZ* fusion plasmids, we utilized reporter constructs that integrate at the Tn*7* attachment site (3), a reporter that uses homology-based recombination to integrate at the native locus of the gene of interest, or a replicating plasmid. Plasmid pMJG561 is a Tn*7*-delivery plasmid with a promoter-less *lacZ* gene that was digested with BamHI and PstI for construction of transcriptional *lacZ* fusions. Plasmid pMJG542 is a Tn*7*-delivery plasmid with a promoter-less *lacZ* gene used for translational fusions when digested with BamHI and HindIII. Plasmid pMJG609 is derived from pMJG561, where the *lacI$^q$*-$P_{tac}$ regulation system drives the expression of *lacZ*. Plasmid pMJG637 is unable to replicate in *Acinetobacter baumannii* and contains a promoter-less *lacZ* gene. Introduction of an appropriate translational fusion and ≈ 1 kb of homology to the target DNA allows for the creation of translational *lacZ* fusions at the desired chromosomal locus, resulting in the *lacZ* fusion reporter being driven by the native promoter of the target gene. Plasmid pMJG779 is a replicating plasmid derived from plasmid pJMP3665 (4) and harbors a promoter-less *lacZ* gene. To construct pMJG779, the promoter-less *lacZ* cassette from pMJG126 (5) was amplified with primers *lacZ_cassette_fwd* and *lacZ_cassette_rev* and the pJMP3665 backbone on an XbaI and HindIII fragment, resulting in pMJG779. Reporter fusions made in pMJG561, pMJG542,

pMJG637, and pMJG779 were created by ITA. For the *dadA* transcriptional fusion in pMJG561, Fwd_*dadA-lacZ* and Rev_*dadA-lacZ_TX* primers were used to amplify a PCR product that starts 500 bp upstream of the *dadA* start codon and ends 16 bp before the *dadA* start codon, the predicted transcription start site for *dadA*. The *dadA* translational fusion was constructed in pMJG542 using primers Fwd_*dadA-lacZ* and Rev_*dadA-lacZ_TL* to amplify a PCR product that starts 500 bp upstream of the *dadA* start codon and encompasses the first twelve amino acids of DadA fused in frame to *lacZ*. The $dadA^{AAG}$ reporter fusion was constructed in pMJG542 using two primer sets: Fwd_*dadA-lacZ* and Rev_$dadA^{AAG}$-*lacZ* to amplify the upstream region of the reporter, and Fwd_$dadA^{AAG}$-*lacZ* with Rev_*dadA-lacZ_TL* to introduce the AAG mutations to the GGA motifs located 1 bp and 6 bp upstream of the *dadA* start codon. The *cycA2* translational fusion in pMJG637 used primers Fwd_*cycA2-lacZ* and Rev_*cycA2-lacZ* to amplify the first 11 codons in *cycA2* through 1 kb upstream which was combined by ITA with pMJG637 linearized with BamHI and HindIII. The $cycA2^{AAG}$ mutant reporter used two primer sets, one to amplify the upstream homology using Fwd_*cycA2-lacZ* and Rev_$cycA2^{AAG}$-*lacZ* and another set to introduce the GGA to AAG mutations located 8 bp and 20 bp upstream of the start of *cycA2* with Fwd_$cycA2^{AAG}$-*lacZ* and Rev_*cycA2-lacZ*. The *cycA2* promoter fusion in pMJG779 used primers Fwd_*cycA-lacZ_TX* and Rev_*cycA2-lacZ_TX* to amplify 25 bp upstream of the *cycA2* start codon (excludes the translation initiation region) through an additional 500 bp upstream of the *cycA2* start codon; this PCR product was inserted into pMJG779 linearized with BamHI.

Replicating plasmids used for complementation and ectopic expression experiments were constructed in plasmid pMJG116 (3). Plasmid pMJG116 carries the coding sequence for mCherry with a strong ribosome binding site. Restriction enzyme digest of this plasmid with NdeI and PstI excises the mCherry CDS but leaves the strong RBS resulting in a suitable cloning site for ectopic expression. The *csrA* coding sequence was amplified using the Fwd_*csrA* and Rev_*csrA* primers, these same primers were used to amplify the RNA binding deficient R44A *csrA* allele using genomic DNA from a strain of AB5075 with this mutation introduced into the chromosome. The *lrp* expression plasmid was created using primers Fwd_*lrp* and Rev_*lrp* to amplify the *lrp* gene. Similarly, the coding sequences for *ABUW_RS18460, ABUW_RS18465, ABUW_RS18470, ABUW_RS18475, ABUW_RS18485, ABUWsRNA106, ABUW_RS18490,* and *ABUW_RS18440*, were all amplified using the respective forward (Fwd) and reverse (Rev) primers as listed in Table S3. A complementation vector for co-expression of *dadA* and *dadX* was created from a PCR product amplified by Fwd_*dadA-dadX* and Rev_*dadA-dadX* that would amplify the *dadA* and *dadX* open reading frames including the 20 bp intergenic region between *dadA* and *dadX.* Individual complementation plasmids were made for *dadA* using the Fwd_*dadA-dadX* and Rev_*dadA,* and for *dadX* using Fwd_*dadX* and the Rev_*dadA-dadX*. The *cycA2* and *cycA1* expression plasmids were created with PCR products amplified from Fwd_*cycA2* and Rev_*cycA2* and Fwd_*cycA1* and Rev_*cycA1*, respectively.

*Whole Genome Sequencing*

Illumina sequencing librariers were prepared using the tagmentation-based and PCR-based Illumina DNA Prep Kit. Custom DNA fragmentation steps were performed to achieve an average target size of 300 bp. Sequencing was performed on an Illumina NovaSeq 6000 sequencer to produce 2x151 paired-end reads. Reads were aligned and mapped to the *A. baumannii* AB5075 genome (CP008706.1) and accessory plasmids (CP008707.1, CP008708.1, and CP008709.1) using Geneious Prime (v 11.0.20.1+1). Single nucleotide polymorphisms (SNPs) were found using the "Find variations/SNPs" function in Geneious with a minimum variant frequency of 0.5, and maximum variant P-value of $10^{-6}$.

*Cloning and Plasmid Construction*

Plasmids used in this study are listed in Supplemental Table S2; oligonucleotide primers are listed in Supplemental Table S3. Molecular cloning of plasmids was performed in E. coli strain DH5a and are described in further detail in the Supplemental methods (see also 3). Briefly, PCR inserts were amplified using KOD Extreme Hot Start DNA polymerase (Sigma Aldrich cat. no. 71975)

according to manufacturer's recommendations. Isothermal assembly (ITA) was facilitated by oligonucleotide primers containing 20-25 nt of homologous bases at the 5¢ ends (1). All plasmid inserts were verified by sequencing at the Iowa State University DNA Facility.

*Complementation*

Genetic complementation was conducting using plasmid pMJG120, an incompatibility group Q plasmid conferring resistance to apramycin and containing an IPTG-inducible promoter ($P_{tac}$) regulated by $LacI^q$. Target genes were amplified and assembled into the plasmid backbone as described above. Successful insertion was confirmed by Sanger Sequencing (Iowa State University DNA Facility). AB5075 and derivatives thereof were made electrocompetent by three washes with 300 mM sucrose. Plasmids were transformed into AB5075 strains by electroporation with a BIORAD Micropulser on setting EC2. Following electroporation, cells were allowed to recover in 900 µL M9-succinate for one hour at 37°C before being plated on M9-succinate plates containing 50 µg/mL apramycin. Transformed cells were subsequently grown in M9-succinate media supplemented with the appropriate antibiotics overnight at 37°C. Cultures were refreshed to an $OD_{600}$ of 0.1 and serially diluted (10-fold dilutions). Five µL of dilutions $10^{-1}$ through $10^{-6}$ were plated onto LB and M9-succinate media containing antibiotic and increasing amounts of IPTG (0, 20 µM, 200 µM and 2 mM respectively) to induce expression from the plasmid. Plates were incubated overnight at 37°C and imaged the next day.

*Construction of AB5075 Mutant strains*

Mutants of A. baumannii AB5075 were constructed essentially as described previously (2, 3). Briefly, derivatives of the pMJG42 allele exchange vector were mobilized into AB5075 through a two-parent mating on LB with an E. coli donor strain (LW264). Transconjugants were selected on LB plates containing tetracycline (10 µg/mL) and chloramphenicol (5 µg/mL) and were subsequently grown on M9-succinate media containing 5% (wt/vol) sucrose to select for colonies that resolved the pMJG42 plasmid backbone. Sucrose resistant colonies were screened by colony PCR and or sequencing to identify clones harboring the desired mutation.

*D-amino acid dehydrogenase (Dad) Assay*

DadA activity was assayed using a modified D-amino acid dehydrogenase protocol (6). Briefly, AB5075 strains and its derivatives were grown overnight in M9-succinate supplemented with 20mM L-alanine at 37°C with shaking at 200 rpm. The following day, the equivalent of 1 $OD_{600}$ unit of cells were collected by centrifugation (10,000 rpm for 1 minute) and washed with 1 mL of 0.1 M Phosphate Buffer (20.2 g/L $Na_2HPO_4 \cdot 7H_2O$, 3.4 g/L $NaH_2PO_4 \cdot H_2O$, pH 7.4) and resuspended 1 mL of fresh 0.1 M Phosphate Buffer. The resulting suspension was incubated on ice for 5 minutes, at which point 100 µL was withdrawn and kept on ice for total protein analysis as described below. The remaining cells were permeabilized with 20 µL toluene and incubated on ice for 20 minutes. Reactions were initiated by adding 100 µL of toluenized cells to 900 µL of 0.1 M phosphate buffer containing 20 mM D-alanine. The reactions were incubated at 37°C for 30 minutes and then 250 µL of 2,4-dinitrophenylhydrazine (0.17 mg/mL in 1.2 N HCl) were added to the reaction tubes. The reactions were briefly vortexed and incubated at room temperature for 15 minutes prior to neutralization with 1 mL of 2.5 N NaOH and an additional 10-minute incubation at room temperature. The $OD_{520}$ values for reach strain were recorded and normalized to total protein content as described below.

*Total Protein Analysis*

The total protein concentration of the collected cells for the D-amino acid dehydrogenase assay was quantified using the Pierce BCA Protein Assay Kit (Thermo Scientific, cat no. 23225) following the manufacturer's recommendations. Prior to the BCA reaction, 100 µL of cells were lysed with 10 µL PopCulture Reagent (Millipore Sigma, cat no. 71092) and

incubated at room temperature for 10 minutes. Standards (generated with bovine serum albumin) and cell lysates were added to the working reagent at a ratio of 1:8, samples were mixed and incubated at 37°C for 30 minutes. The samples were cooled to room temperature and the $OD_{562}$ was measured on a SpectaMax190 microplate reader (Molecular Devices). Protein concentration in the lysate was determined based on the control standard curve and used to normalize the Dad assay results.

*RNA Immunoprecipitation (RIP)*
For RIP-qPCR, cells from 80 mL of triplicate exponentially growing cultures ($OD_{600} \approx 0.5$; 40 OD units) of the AB5075 CsrA-V strain were collected by centrifugation. Cell pellets were washed thrice with PBS and subjected to ultraviolet radiation to induce crosslinking. Cross-linked cells were lysed by bead beating. Immunoprecipitation (IP) was performed on cells lysates with murine monoclonal anti-VSV-G antibodies (Sigma Alrich cat. no. SAB4200695). Cells collected from a second set of CsrA-V cultures were subjected to the IP without antibody to serve as a no-antibody/mock control. Following IP, proteins were digested with Proteinase K (ThermoFisher cat. no. EO0491) and RNA was extracted with TriReagent LS according to the manufacturer's recommendations; GlycoBlue (Ambion, cat. no. AM9515) was added to aid in RNA precipitation. The resulting RNA was converted to cDNA and used for RT-qPCR as described above. The relative abundance of the target genes was normalized to a control transcript, *recA*, and the enrichment of transcripts in the IP samples relative to the no antibody control samples are reported. RIP and RT-qPCR experiments were independently repeated twice on biological triplicate cultures. Data are depicted as the mean relative enrichment from all six RIP replicates with error bars representing one standard deviation of the mean.

*Western blotting*
For the CsrA-V western blot, overnight cultures of AB5075 CsrA-V encoding a defective Cas9 allele (dCas9) at the Tn*7* attachment site harboring either a control guide plasmid or a single guide targeting *csrA*, were refreshed in LB containing apramycin (25 µg/mL) at a starting density of 0.01 $OD_{600}$ units. The cultures were grown in the absence of induction with shaking at 37°C. Western blot samples were collected at three hours ($OD_{600} \approx 0.2$-$0.4$) and at four hours ($OD_{600} \approx 1$-$1.5$); at each time point 1 mL of cells were pelleted. Cell pellets were dissolved in 1x LDS Sample Buffer (Thermo Fisher cat. no. NP0007), boiled for 10 minutes, briefly sonicated, and resolved through a 12% Bis-Tris NuPAGE gel (Thermo Fisher cat no. NP0321) in 1x MES running buffer (Thermo Fisher cat. no. NP0002). Proteins were transferred to a Immobilon-PSQ polyvinylidene fluoride membrane (EMD Millipore cat. no. ISEQ08100) using an XCell-II Blot Module (Thermo Fisher). Membranes were fixed with methanol and blocked overnight in a 1:5 dilution of blocking buffer (LI-COR cat no. 071-70001) in PBS before being probed for 1 hour with rabbit anti-VSVG (1:3,333 dilution in diluted blocking buffer, Sigma Aldrich cat no. V4888) and mouse anti-RpoA antibodies (1:10,000 dilution in diluted blocking buffer, BioLegend cat no. WP003). The membranes were washed four times with wash buffer (1x PBS + 0.05% Tween; Sigma Aldrich cat no. P1379), blocked again for 20 minutes, and probed with goat anti-rabbit IgG secondary antibody conjugated with the nearinfrared (IR) dye 800 and goat anti-mouse IgG secondary antibody conjugated with the near-IR dye 700 (1:15,000 dilutions in wash buffer, Azure Biosystems cat. no. AC2134 and AC2129 respectively) for 1 hour. The membrane was washed four times with wash buffer and two times with 1x PBS, dehydrated with methanol, and imaged using an Azure Sapphire Imaging System (Azure Biosciences). Western blot

experiments were repeated independently two times, and the results from a single representative experiment are shown.

*MIC testing*

For vancomycin, meropenem, and fosfomycin, additional antimicrobial resistance testing was also performed by E-strip (Liofilchem cat. no. 92057, 92057, and 920781, respectively). Strains were grown overnight in M9-succinate and 50 μL of culture was added to 5 mL of molten M9-succinate agar (0.75% agar). The molten agar was poured onto a solid M9-succinate plate to create a bacterial overlay. The agar overlay was allowed to solidify for 20 minutes at room temperature before adding the antibiotic E-strip. Plates were incubated overnight at 37°C and MIC was determined by the concentration where the zone of inhibition intersected with the E-strip.

**Supplemental Figures**

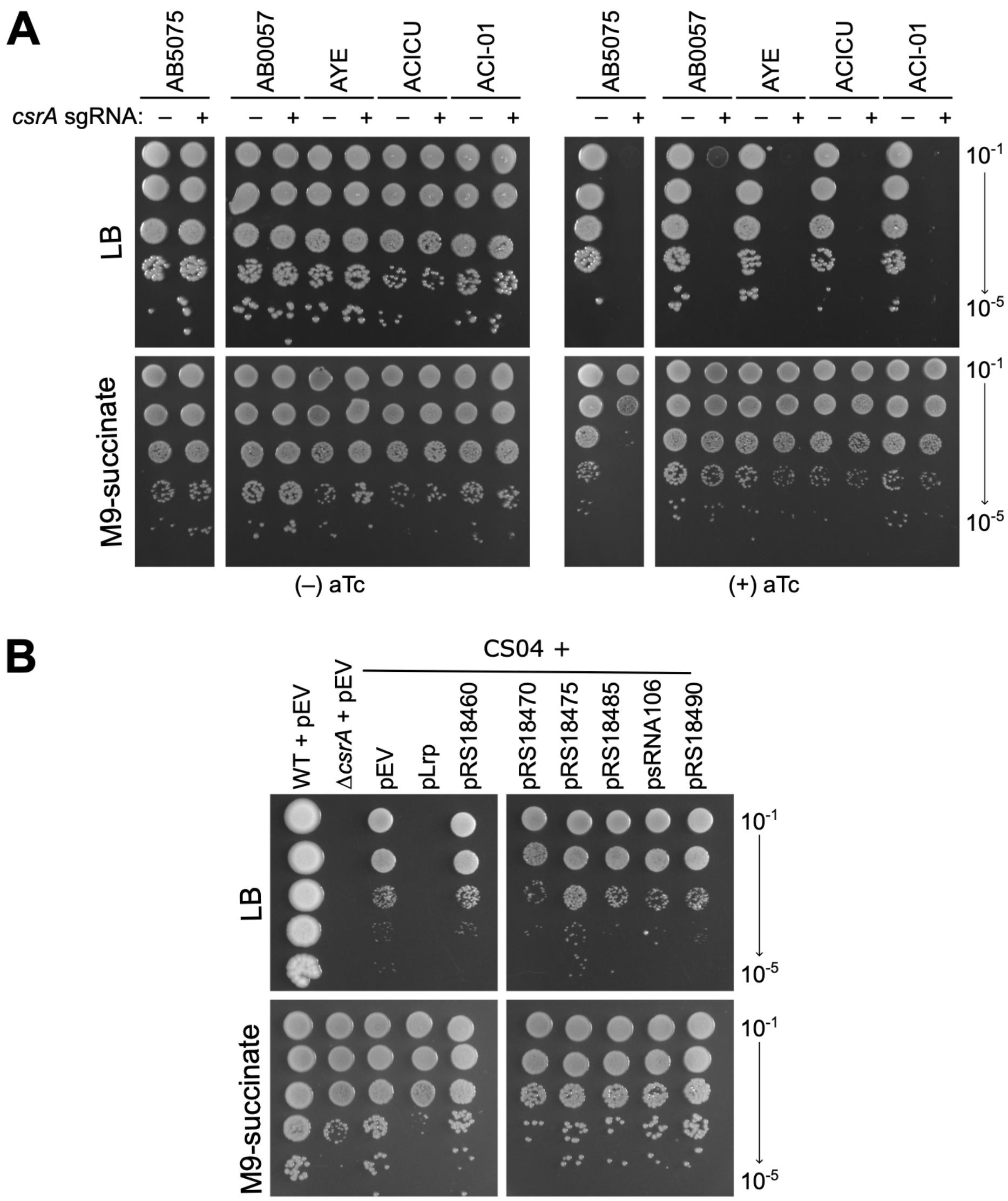


**Fig. S1. Depletion of *csrA* prevents growth of *A. baumannii* strains on LB and ectopic expression of Lrp restores the *csrA* phenotype in suppressor strain CS04**. Serial dilutions of (A) AB5075,7, AYE, ACICU, and ACI-01 containing a defective Cas9 (*dCas9*) at the Tn*7* attachment site transformed with a non-targeting single guide RNA (sgRNA) or an sgRNA targeting *csrA* on LB (top) and M9-succinate (bottom) with and without 100 ng/mL aTc (B) AB5075 (WT), Δ*csrA,* and CS04 harboring the indicated plasmids were plated on LB (top) and M9-succinate (bottom). pEV, empty vector.

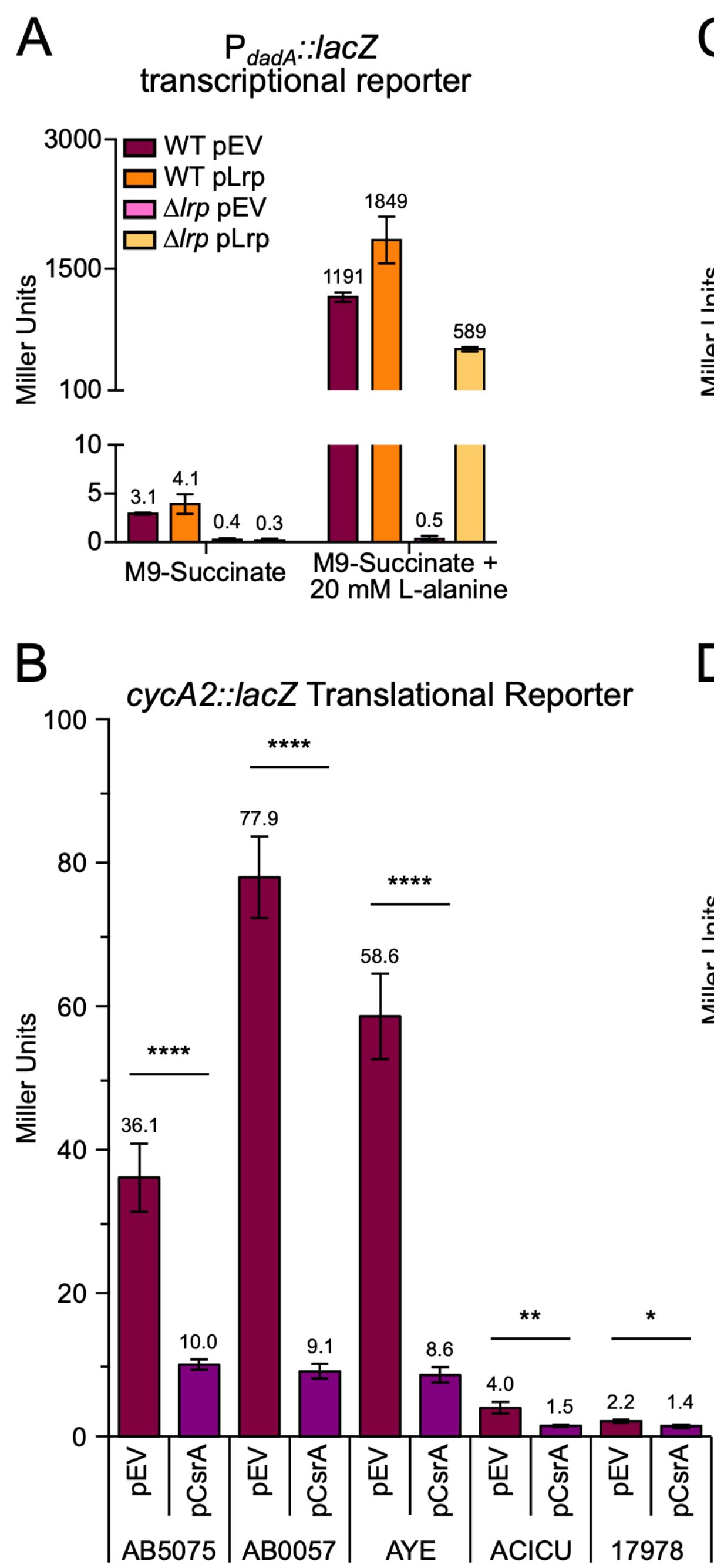


**Fig. S2. Effect of *csrA* and *lrp* on *lacZ* reporters and conservation analysis for regulation of *cycA2* in other *A. baumannii* strains**. (A) Beta-galactosidase assay results of $P_{dadA}$*::lacZ* transcriptional reporter at the Tn7 attachment site in wild-type (WT) AB5075 or Δ*lrp* transformed with empty vector control (pEV) or *lrp* expression plasmid (pLrp) in M9-succinate and M9succinate supplemented with 20 mM L-alanine. (B) Miller units of the *cycA2::lacZ* translational reporter in WT AB5075, AB0057, AYE, ACICU, and 17978 cells transformed with pEV or pCsrA. (C) *lrp::lacZ* translational reporter activity in WT AB5075 and Δ*csrA* transformed with pEV and pCsrA in M9-succinate supplemented with 20 mM L-alanine. (D) $P_{lrp}$*::lacZ* reporter activity in WT AB5075 or Δ*csrA* transformed with pEV or pCsrA in M9-succinate with 20 mM L-alanine. Statistical analysis in (B) performed as two-tailed t-test with Bonferroni correction post hoc test to account for multiple comparisons; *, $p < 0.05$; **, $p < 0.01$; ***, $p < 0.001$; ****, $p < 0.0001$.

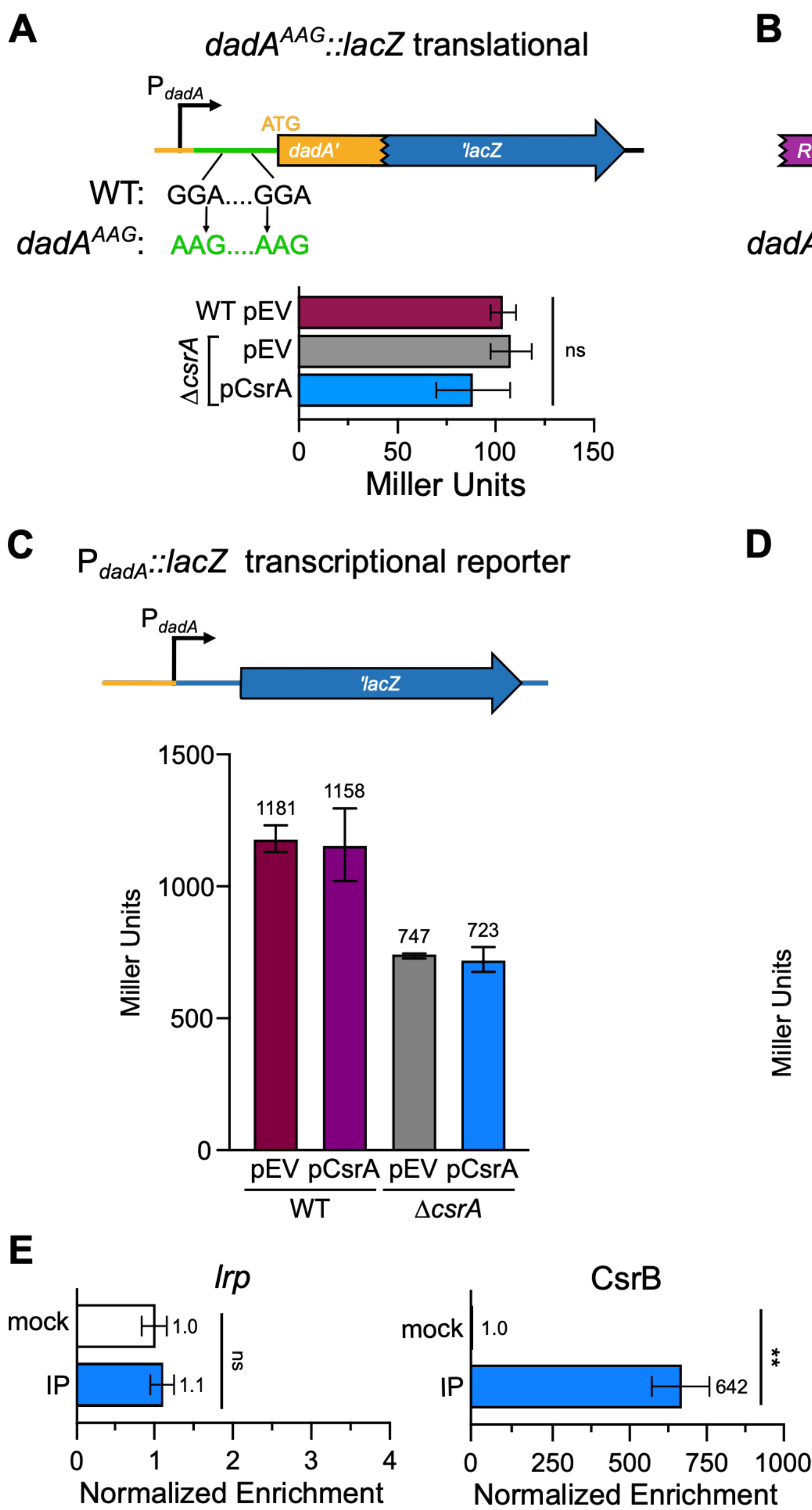


**Fig. S3. DadA and CycA2 are regulatory targets of CsrA**. Beta-galactosidase assay results from (A) *dadA*$^{AAG}$*::lacZ and* (B) *cycA*$^{AAG}$*::lacZ* translational reporters and (C) P$_{dadA}$*::lacZ and (D) P*$_{cycA2}$*::lacZ* transcriptional reporters in WT AB5075 or Δ*csrA* transformed with indicated plasmids. Beta-galactosidases assays performed in biological triplicate from exponentially growing cells cultured in M9-succinate with 20 mM L-alanine. Schematics depict reporter constructs. In (D), grey brackets indicate fold change in activity for the *lacZ* control vector and P$_{cycA2}$*::lacZ* vector for the WT and Δ*csrA* strains; TIR, translation initiation region. (E) RIP-qPCR analysis with CsrA-V for the indicated transcripts. Cultures (n = 3) grown in LB to exponential phase prior to IP; mock, IP performed with no antibody; IP, IP performed with anti-VSV-G antibodies. Significance assessed by one-way ANOVA with Dunnett's post-hoc test to correct for multiple comparisons (AD) or two-tailed t-test (E). Asterisks indicate significant differences from WT with pEV for βgalactosidase assays and from the mock control sample for RIP-qPCR; *, $P < 0.05$; **, $P < 0.01$; ***, $P < 0.001$; ****, $P < 0.0001$; ns, not significant.

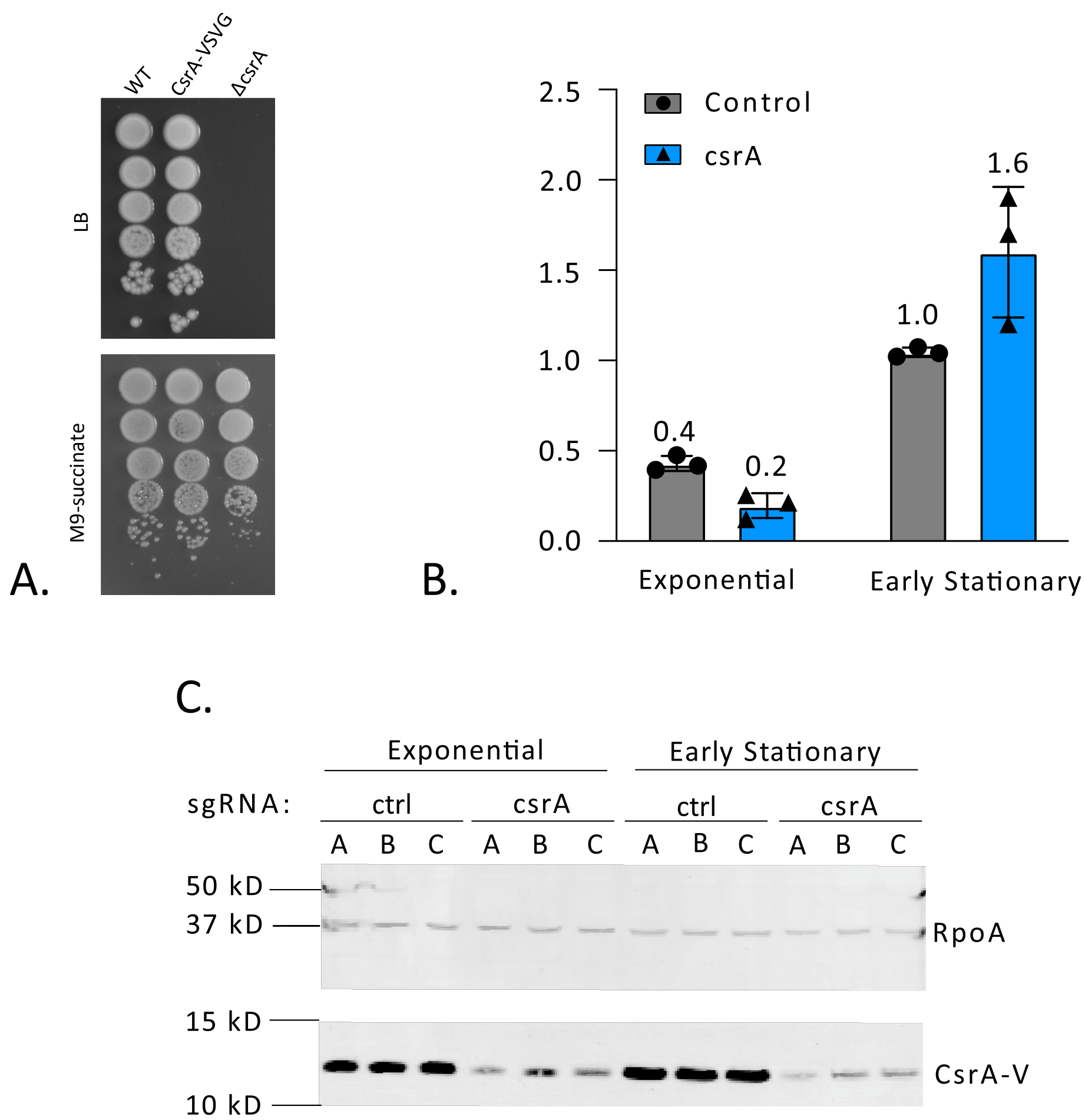


**Fig. S4. Validation of CsrA-V and *csrA* CRISPRi knockdown.** (A) Serial dilutions of wild-type AB5075 (WT), CsrA-VSVG, and Δ*csrA* plated on LB (top) and M9-succinate (bottom). (B) $OD_{600}$ values of CRISPRi samples containing a control or *csrA* targeting guide RNA collected from exponential and early stationary phase cultures grown in LB broth. (C) Western blot of CRISPRi samples containing a control guide RNA (targeting *mCherry*) or a *csrA* targeting guide RNA, as indicated. For C, CsrA-V blot was detected using anti-VSV-G antibodies, anti-RpoA antibodies were used to detect RpoA as a loading control. The migration of size standards are indicated to the left.

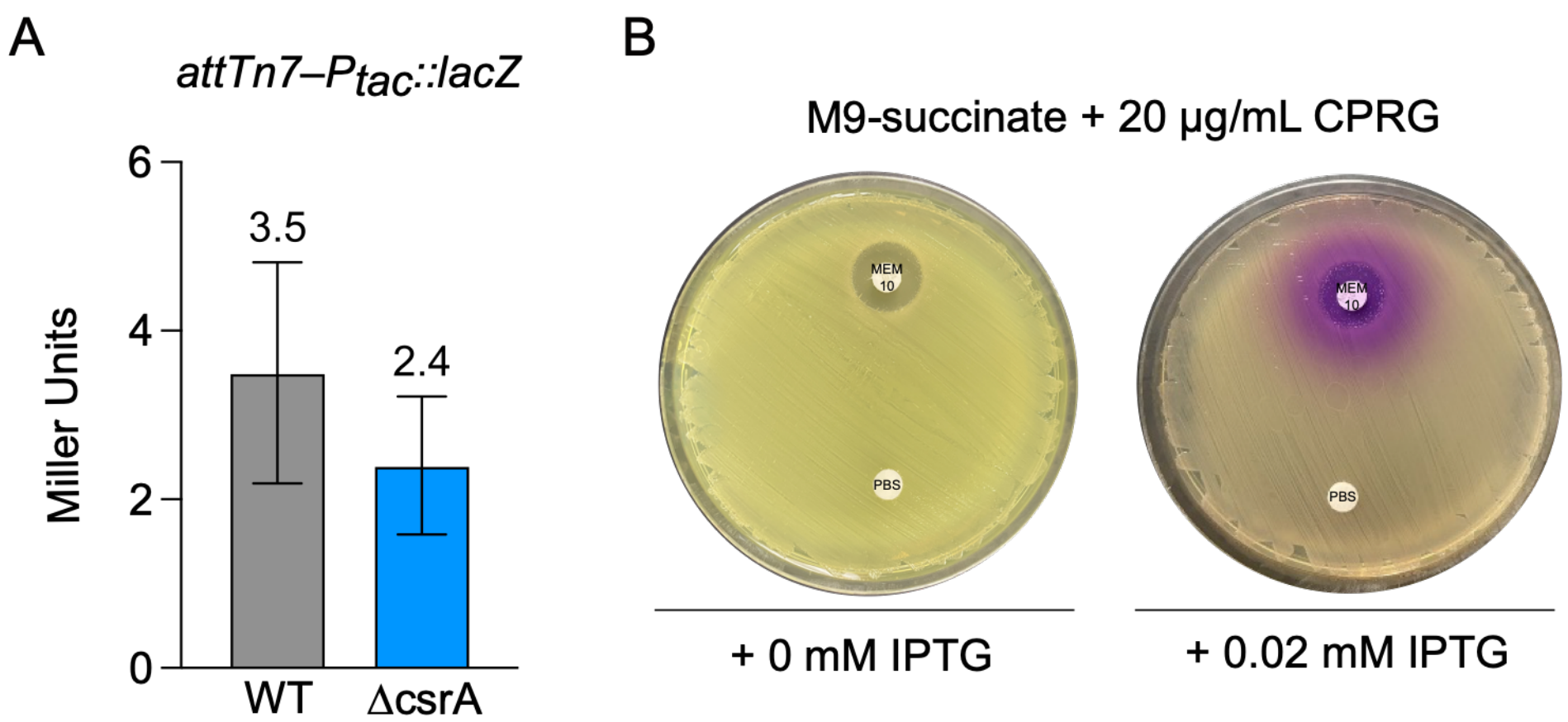


**Fig. S5. CPR production does not occur in the absence of cell wall targeting antibiotics for wild-type AB5075.** (A) Beta-galactosidase assay results for strains harboring *$P_{tac}$::lacZ* transcriptional reporter in wild-type AB5075 and Δ*csrA.* Cultures (n = 3) were grown to exponential phase in in M9-succinate without IPTG and assayed for beta-galactosidase activity as described in the main text. (B) Wild-type AB5075 containing $P_{tac}$ *lacZ* plated on M9-succinate agar plates containing 20 µg/mL chlorophenyl red-b-D-galactopyranoside (CPRG) and the indicated amount of IPTG in the presence of meropenem discs (MEM 10, top discs) or discs soaked in PBS (bottom discs).

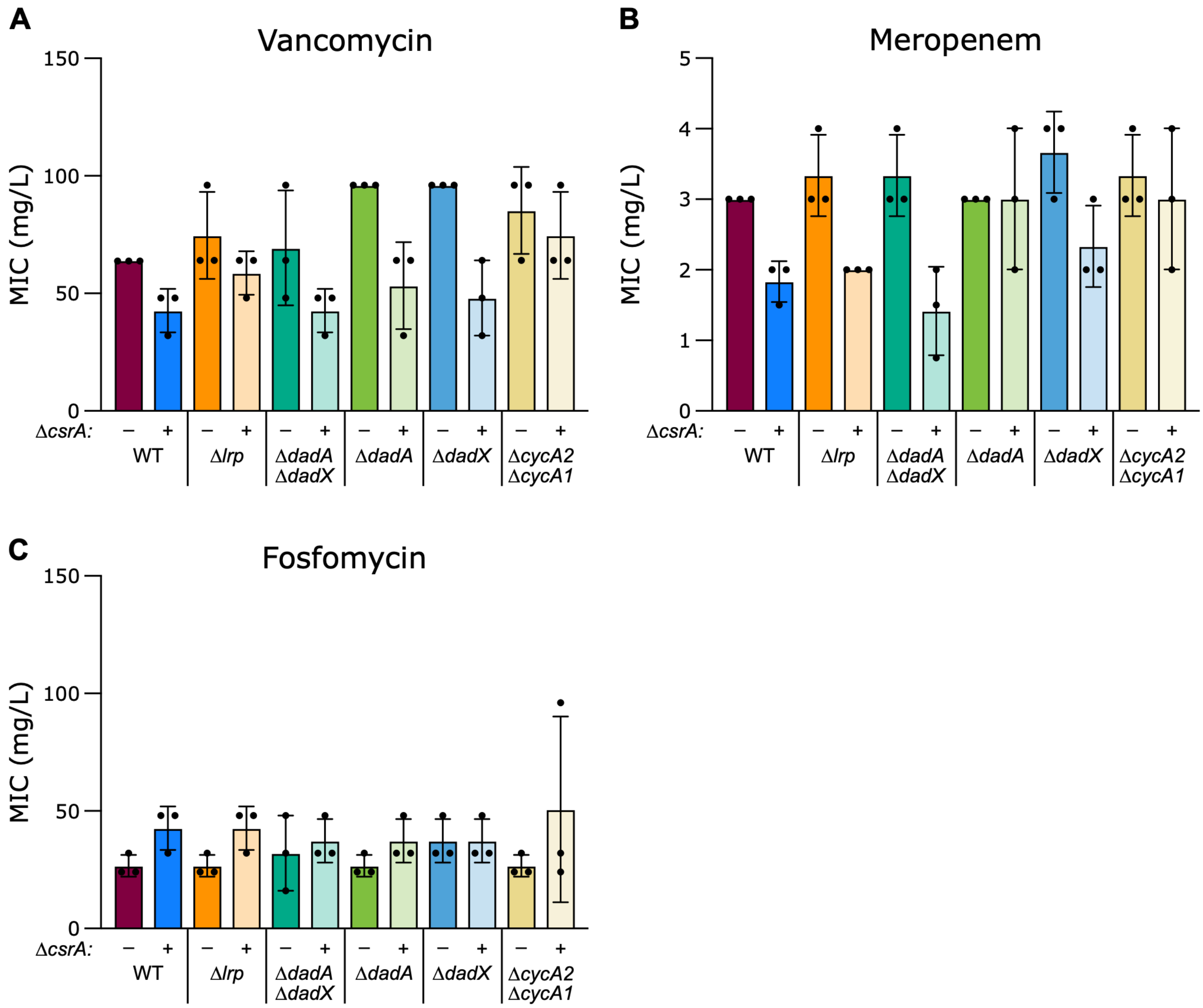


**Fig S6. Antimicrobial sensitivity testing of strains lacking *dad* locus genes.** Graphs depict antimicrobial sensitivity testing for the indicated strains with or without the Δ*csrA* mutation for (A) vancomycin, (B) meropenem, and (C) fosfomycin. MICs determined in triplicate using e-test strips on M9-succinate agar. Data points indicate MIC values obtained from three independent experiment runs.

## Supplemental Tables

**Table S1.** Bacterial Strains used in this study

| Strain | Description | Reference |
|---|---|---|
| *Escherichia coli* | | |
| DH5α F′-*lacI*$^{q}$ | cloning strain | Lab Stock |
| LW264 | Donor strain for mobilizing plasmids into *A. baumannii* | (2) |
| SM10 lpir$^{+}$ pTNS3 | Tn*7* mating helper plasmid | Lab Stock |
| Top10 pRK2013 | Tn*7* mating helper plasmid | Lab Stock |
| *Acinetobacter baumannii* | | |
| 17978 | *A. baumannii* strain ATCC 17978 | Lab Stock |
| AB0057 | *A. baumannii* strain AB0057 | (7) |
| AYE | *A. baumannii* strain AYE | (8) |
| ACICU | *A. baumannii* strain ACICU | (9) |
| ACI-01 | *A. baumannii* clinical isolate | (10) |
| AB5075 | AB5075-UW, multidrug resistant isolate | (11) |
| RLS12 | AB5075, *csrA-VSVG* | This study |
| RLS13 | AB5075 Δ*csrA* | This study |
| RLS14 | AB5075 Δ*csrA* | This study |
| RLS33 | AB5075 Δ*lrp* | This study |
| RLS36 | AB5075 Δ*lrp* Δ*csrA* | This study |
| RLS62 | AB5075 Δ*dadA* Δ*dadX* | This study |
| RLS65 | AB5075 Δ*dadA* Δ*dadX* Δ*csrA* | This study |
| RLS86 | AB5075 Δ*ABUW_RS18440* | This study |
| RLS87 | AB5075 ΔABUW_RS18440 Δ*csrA* | This study |
| RLS91 | AB5075 Δ*dadA* | This study |
| RLS93 | AB5075 Δ*dadX* | This study |
| RLS95 | AB5075 Δ*dadX* Δ*csrA* | This study |
| RLS98 | AB5075 *dadA-lacZ* transcriptional fusion at Tn*7* site (*dadA::lacZ*$^{TX}$) | This study |
| RLS99 | AB5075 *dadA-lacZ* translational fusion at Tn*7* site (*dadA::lacZ*$^{TL}$) | This study |
| RLS109 | AB5075 Δ*lrp dadA::lacZ*$^{TX}$ | This study |
| RLS114 | AB5075 Δ*cycA2* | This study |
| RLS135 | AB5075 Δ*dadA* Δ*csrA* | This study |
| RLS180 | AB5075 *cycA2::lacZ*$^{TL}$ | This study |
| RLS183 | AB5075 Δ*lrp cycA2::lacZ*$^{TL}$ | This study |
| RLS192 | AB5075 Δ*cycA1* | This study |
| RLS202 | AB5075 Δ*cycA1* Δ*cycA2* | This study |
| RLS210 | AB5075 Δ*cycA1* Δ*cycA2* Δ*csrA* | This study |

| | | |
|---|---|---|
| RLS238 | AB5075 *dCas9* at Tn*7* site | (3) |
| RLS239 | AB5075 *csrA-VSVG dCas9* at Tn*7* site | This study |
| RLS270 | AB5075 Δ*csrA dadA::lacZ*$^{TX}$ | This study |
| RLS273 | AB5075 Δ*csrA dadA::LacZ*$^{TL}$ | This study |
| RLS282 | AB5075 *dadA*$^{AAG}$*::lacZ*$^{TL}$ | This study |
| RLS284 | AB5075 Δ*csrA cycA2::lacZ* | This study |
| RLS303 | AB5075 *cycA2*$^{AAG}$*::lacZ* | This study |
| RLS319 | AB5075 $P_{tac}$*::lacZ* at Tn*7* site | (3) |
| RLS334 | AB5075 Δ*csrA* $P_{tac}$*::lacZ* at Tn*7* site | This study |
| RLS341 | AB5075 Δ*csrA cycA2*$^{AAG}$*::lacZ* | This study |
| RLS367 | AB5075 *lrp-lacZ* transcriptional fusion at Tn*7* site (*lrp::lacZ*$^{TX}$) | This study |
| RLS369 | AB5075 *lrp-lacZ* translational fusion at Tn*7* site (*lrp::lacZ*$^{TL}$)) | This study |
| RLS540 | AB5075 Δ*csrA lrp::lacZ*$^{TX}$ | This study |
| RLS542 | AB0057 *cycA2::lacZ*$^{TL}$ | This study |
| RLS544 | AYE *cycA2::lacZ*$^{TL}$ | This study |
| RLS546 | ACICU *cycA2::lacZ*$^{TL}$ | This study |
| RLS548 | AB17978 *cycA2::lacZ*$^{TL}$ | This study |
| RLS564 | AB5075 Δ*csrA lrp::lacZ*$^{TL}$ | This study |
| VH191 | AYE dCas9 at Tn*7* site | (12) |
| VH195 | ACICU dCas9 at Tn*7* site | (12) |
| VH199 | ACI-01 dCas9 at Tn*7* site | (12) |

**Table S2.** Plasmids used in this study.

| Plasmids to introduce mutations to *A. baumannii* chromosome | | Source |
|---|---|---|
| pMJG42 | Allele exchange vector | (13) |
| pMJG381 | Deletion of *csrA (ABUW_RS13350)* open reading frame | This study |
| pRLS08 | Deletion of *lrp* (*ABUW_RS18455)* open reading frame | This study |
| pRLS41 | Deletion of *dadA (ABUW_RS18450)* and *dadX (ABUW_RS18445)* open reading frames | This study |
| pRLS48 | Deletion of *ABUW_RS18440* open reading frame | This study |
| pRLS50 | Deletion of *dadA* (*ABUW_RS18450)* open reading frame | This study |
| pRLS51 | Deletion of *dadX* (*ABUW_RS18445)* open reading frame | This study |
| pRLS54 | Deletion of *cycA2* (*ABUW_RS18435)* open reading frame | This study |
| pRLS67 | Deletion of *cycA1 (ABUW_RS18430)* and *cycA2 (ABUW_RS18435)* open reading frame | This study |
| pRLS68 | Deletion of *cycA1* (*ABUW_RS18430)* open reading frame | This study |
| pRLS69 | Deletion of *dadA (ABUW_RS18250), dadX (ABUW_RS18445), ABUW_RS18440, cycA2 (ABUW_RS18435)* and *cycA1 (ABUW_RS1843)* open reading frames | This study |
| | | |
| Complementation plasmids | | |
| pMJG120 | Empty vector, expression controlled by *lacI^q^-P~tac~* → $lacI^q$-$P_{tac}$ | (2) |
| pRLS06 | pMJG120 with *csrA* (*ABUW_RS13350*) under control of $lacI^q$-$P_{tac}$ | This study |
| pRLS09 | pMJG120 with *lrp (ABUW_RS18455)* under control of $lacI^q$-*Ptac* | This study |
| pRLS10 | pMJG120 with *ABUW_RS18460* under control of $lacI^q$-$P_{tac}$ | This study |
| pRLS11 | pMJG120 with *ABUW_RS18470* under control of $lacI^q$-$P_{tac}$ | This study |
| pRLS12 | pMJG120 with *ABUW_RS18475* under control of $lacI^q$-$P_{tac}$ | This study |
| pRLS13 | pMJG120 with *ABUW_RS18485* under control of $lacI^q$-$P_{tac}$ | This study |
| pRLS14 | pMJG120 with sRNA106 (formerly *ABUW_3797*) under control of $lacI_q$-$P_{tac}$ | This study |
| pRLS15 | pMJG120 with *ABUW_RS18490* under control of $lacI^q$-$P_{tac}$ | This study |
| pRLS17 | pMJG120 with *csrA R44A* under control of $lacI^q$-$P_{tac}$ | This study |
| pRLS31 | pMJG120 with *ABUW_RS18440* under control of $lacI^q$-$P_{tac}$ | This study |
| pRLS32 | pMJG120 with *cycA2 (ABUW_RS18435)* under control of $lacI^q$-$P_{tac}$ | This study |
| pRLS57 | pMJG120 with *dadA (ABUW_RS18450)* and *dadX (ABUW_RS18455)* under control of $lacI^q$-$P_{tac}$ | This study |
| pRLS62 | pMJG120 with *dadA (ABUW_RS18450)* under control of $lacI^q$-$P_{tac}$ | This study |
| pRLS63 | pMJG120 with *dadX (ABUW_RS18455)* under control of $lacI^q$-$P_{tac}$ | This study |
| pRLS64 | pMJG120 with *cycA1 (ABUW_RS18430)* under control of $lacI^q$-$P_{tac}$ | This study |
| | | |
| Tn7 Integration Plasmids | | |
| pMJG542 | Tn*7*-delivery plasmid for *lacZ* translational fusions ($lacZ^{TL}$) | This study |
| pMJG561 | Tn*7*-delivery plasmid for *lacZ* transcriptional fusions ($lacZ^{TX}$) | (3) |
| pMJG609 | Tn*7*-delivery plasmid for *lacZ* under $lacI^q$-$P_{tac}$ expression control | (3) |

| | | |
|---|---|---|
| pRLS52 | Tn*7* plasmid for *dadA::lacZ* transcriptional fusion | This study |
| pRLS53 | Tn*7* plasmid for *dadA::lacZ* translational fusion | This study |
| pRLS59 | Tn*7* plasmid for $dadA^{AAG}$*::lacZ* translational fusion | This study |
| | | |
| Single-cross LacZ integration plasmids | | |
| pMJG637 | Delivery plasmid for insertion of *lacZ* translational fusions | This study |
| pRLS65 | *cycA2::lacZ* translational fusion | This study |
| pRLS71 | $cycA2^{AAG}$*::lacZ* translational fusion | This study |
| | | |
| Replicating LacZ plasmids | | |
| pMJG779 | Empty vector for replicating *lacZ* promoter fusions | This study |
| pRLS123 | *cycA2::lacZ* transcriptional fusion | This study |
| | | |
| CRISPRi plasmids | | |
| pMJG440 | Control sgRNA plasmid | (3) |
| pMJG492 | pMJG440 + *csrA* sgRNA 1 | This study |
| pMJG498 | pMJG440 + *csrA sgRNA 2* | This study |

**Table S3.** Primers used in this study.

| ***Deletion Primers*** | | |
|---|---|---|
| RLS13 | Ex_Fwd_*lrp* | **AGCCCGGGGGATCCACTAGT**AAAAGCAGGGTCAA CAATAGGAATCGT |
| RLS14 | Ex_Rev_*lrp* | **AGGGCCGCGGTGGCGGCCGC**CCGGTTTTGTACT TTTAACCGGTAT |
| RLS15 | In_Fwd_*lrp* | TCTATGGCGGCCGCATAAAGCGAGCATTACTGAAC TCGCT |
| RLS16 | In_Rev_*lrp* | CTTTATGCGGCCGCCATAGATATTTCCTCTTTTTTT AAAATTCTCGCGAG |
| RLS98 | Ext_Fwd_*ABUW_RS18440* | **ATATCGAATTCCTGCAGCCCGGGGGATCCACTAG** TTGTGATCCCACGGTGTTACCG |
| RLS99 | Ex_Rev_*ABUW_RS18440* | **GTGAGAGGGCCGCGGTGGCGGCCGC**GGACATGG AATTGAACGTGTCTATGAAGCA |
| RLS100 | In_Fwd_*ABUW_RS18440* | GAATTAGCGGCCGCACATTCGGATTTCCTTATACCT GATTA |
| RLS101 | In_Rev_*ABUW_RS18440* | CGAATGTGCGGCCGCTAATTCGGCTTTACTCCAAA AACGAT |
| RLS102 | Ex_Fwd_*dadA-dadX* | **CCTGCAGCCCGGGGGATCCACTAGT**AAGGAGGTT AAGACCCATCACAAACAACA |
| RLS140 | Ex_Rev_*dadA-dadX* | **GTGAGAGGGCCGCGGTGGCGGCCGC**ACCTCACC AAGTATCCACCGTCTTT |
| RLS103 | In_Fwd_*dadA-dadX* | TCCTTAGCGGCCGCACATTTCCATTCCCTCACGCA CACCAGTTTA |
| RLS104 | In_Rev_*dadA-dadX* | AAATGTGCGGCCGCTAAGGAAATCCGAATGTCTAA TTCAGATATA |
| RLS136 | Ex_Fwd_*cycA2* | **CCTGCAGCCCGGGGGATCCACTAGT**AACAGACCA CCATGCGACCAG |
| RLS137 | Ex_Rev_*cycA2* | **GTGAGAGGGCCGCGGTGGCGGCCGC**GCGGCATC ATGCTTTATGGCA |
| RLS138 | In_Fwd_*cycA2* | TAAAACTTAGCGGCCGCACATATTAAAATTCCTTTT TGAATTCCTTTGTTAAGATC |
| RLS139 | In_Rev_*cycA2* | GAATTTTAATATGTGCGGCCGCTAAGTTTTATTAAA AAGCCAATCGAACATGATTG |
| RLS163 | In_Fwd_*dadX* | CCTTAGCGGCCGCACATGATTACTTTCCTAACGTC CGTTAAGCCG |
| RLS164 | In_Rev_*dadX* | ATCATGTGCGGCCGCTAAGGAAATCCGAATGTCTA ATTCAGATAT |
| RLS165 | Ex_Fwd_*dadA* | **CCTGCAGCCCGGGGGATCCACTAGT**ACCACTTCA CTACCAACTTTGGCAC |
| RLS166 | In_Fwd_*dadA* | CGTTAGCGGCCGCACATTTCCATTCCCTCACGCAC ACCAGTTTATT |
| RLS167 | In_Rev_*dadA* | AAATGTGCGGCCGCTAACGGACGTTAGGAAAGTAA TCATGCCTCGT |
| RLS193 | Ex_Fwd_*cycA1*/Ex_Fwd_*cycA1cycA2* | **CCTGCAGCCCGGGGGATCCACTAGT**ACCAACGCA AATTCATGCACGATTGT |
| RLS194 | Ex_Rev_*cycA1* | **GTGAGAGGGCCGCGGTGGCGGCCGC**GTAACACC GTGGGATCACATTCGTG |
| RLS195 | In_Fwd_*cycA1* | TAATTAGCGGCCGCACATATTAGAAGTCCTTTTTAT ATTTGCTTA |

| | | |
|---|---|---|
| RLS196 | In_Rev_*cycA1* | ATATGTGCGGCCGCTAATTAACCTGTCTCGATTTACCAGAAAGCC |
| RLS199 | In_Fwd_*cycA2A1* | GTTAATTAGGCGGCCGCACATATTAAAATTCCTTTTTGAATTCCTTTGTTA |
| RLS200 | In_Rev_*cycA2A1* | TTAATATGTGCGGCCGCCTAATTAACCTGTCTCGATTTACCAGAAAGCCAA |

| ***Primers for construction of lacZ fusions*** | | |
|---|---|---|
| RLS156 | Fwd_*dadA-lacZ* | **CATGAGCTCGAATTCCCGGGGATCC**TAATAGATCTCCTAACAAGCGACGATATGCACT |
| RLS157 | Rev_*dadA-lacZ_TX* | **CGGGATCGCTAGTTAGTTACTGCAG**ACACCAGTTTATTTCACTAGTATATTTCTGCTTCA |
| RLS158 | Rev_*dadA-lacZ_TL* | **GTTGTAAAACGACGGGAGCAAGCTT**TCCAATAACGCCGCTACCTAGTACAATT |
| RLS179 | Fwd_*dadA*$^{AAG}$-*lacZ* | AAGCAGAAATATACTAGTGAAATAAACTGGTGTGCGTGAGAAGATAAGAATGCGCGTA |
| RLS180 | Rev_*dadA*$^{AAG}$-*lacZ* | ACGCACACCAGTTTATTTCACTAGTATATTTCTGCTT |
| RLS187 | Fwd_*cycA2-lacZ* | **TGGGGATCGGAATTCCCGGGGATCC**CAGATTACCCAACCCATAGTATTGCGGATT |
| RLS188 | Rev_*cycA2-lacZ_TL* | **GTTGTAAAACGACGGGAGCAAGCTT**ATTTTCCGTATCTGAATGACGAGCCGT |
| RLS248 | Fwd_*cycA2*$^{AAG}$-*lacZ_TL* | AATTTTCGCCATAGGTTGCTGATCTTAACAAAAAGATTCAAAAAAAGATTTTAATATGAC |
| RLS249 | Rev_*cycA2*$^{AAG}$-*lacZ_TL* | GTTAAGATCAGCAACCTATGGCGAAAATT |
| RLS261 | Fwd_*cycA2-lacZ_TX* | **ATCGGAATTCCCGGGGATCC**ATGTCTAATTCAGATATACAAAAGATTAACACTAACGA |
| RLS343 | Rev_*cycA2-lacZ_TX* | **CCGCTTTCATCGGTTGTCCGGATCC**TGTTAAGATCAGCAACCTATGGCGAAAATTGA |

| ***Primers for complementation vectors*** | | |
|---|---|---|
| RLS10 | Fwd_*csrA lacI*$^{q}$-$P_{tac}$ | **AATTCTTTAAGAAGGAGATATACAT**ATGCTGATTCTGACCCGCCGTGT |
| RLS04 | Rev_*csrA lacI*$^{q}$-$P_{tac}$ | **ACAGCCAAGCTTGCATGCCTGCAGC**TAACGATTGAAGTTTTTCTGTGCATAATTATCAT |
| RLS17 | Fwd_*lrp lacI*$^{q}$-$P_{tac}$ | **TTTAAGAAGGAGATATACAT**ATGCGCCCCTTAGATCGTATAGATCG |
| RLS18 | Rev_*lrp lacI*$^{q}$-$P_{tac}$ | **CCGCCAAAACAGCCAAGCTT**TTACTTGCTCACATCGAGATATAGAGTTTCTTTCA |
| RLS19 | Fwd_*ABUW_RS18460 lacI*$^{q}$-$P_{tac}$ | **TTTAAGAAGGAGATATACAT**ATGTCTTTAGAAAAGAATATTGAACGTTTACAGTCG |
| RLS20 | Rev_*ABUW_RS18460 lacI*$^{q}$-$P_{tac}$ | **CCGCCAAAACAGCCAAGCTT**TTAGAAATATTTTAAAATCGAAATATGCTTGTTTTGATGT |
| RLS21 | Fwd_*ABUW_RS18465 lacI*$^{q}$-$P_{tac}$ | **TTTAAGAAGGAGATATACAT**ATGCGACACAAATTAGGATTGTTGTCTTCG |
| RLS22 | Rev_*ABUW_RS18465 lacI*$^{q}$-$P_{tac}$ | **CCGCCAAAACAGCCAAGCTT**TTAAAACTTCCACTGTGCCGAAATTGAGG |
| RLS23 | Fwd_*ABUW_RS18470 lacI*$^{q}$-$P_{tac}$ | **TTTAAGAAGGAGATATACAT**ATGAACTTATCTCAAAAACAAATTGCAGTACGG |

| | | |
|---|---|---|
| RLS24 | Rev_*ABUW_RS18470 lacI*$^q$-$P_{tac}$ | **CCGCCAAAACAGCCAAGCTT**TTAGAACATACCATTTTCATTGACGAGCGG |
| RLS25 | Fwd_*ABUW_RS18475 lacI*$^q$-$P_{tac}$ | **TTTAAGAAGGAGATATACAT**ATGAACCGCGATTACGAACAATTTCC |
| RLS26 | Rev_*ABUW_RS18475 lacI*$^q$-$P_{tac}$ | **CCGCCAAAACAGCCAAGCTT**TTACTGCAACAGGTCATCTTCTTCGT |
| RLS27 | Fwd_*ABUW_RS18485 lacI*$^q$-$P_{tac}$ | **TTTAAGAAGGAGATATACAT**ATGGAGAATAAAAATCAGTGTCGCCAATG |
| RLS28 | Rev_*ABUW_RS18485 lacI*$^q$-$P_{tac}$ | **CCGCCAAAACAGCCAAGCTT**TTATCGTTTTTTATTCATTTCAGCTAAAAGATCGAGC |
| RLS29 | Fwd_A*BUW_3797 lacI*$^q$-$P_{tac}$ | **TTTAAGAAGGAGATATACAT**TTGCCTAATGGCGTAAAAATTCCCAAAAA |
| RLS30 | Rev_*ABUW_3797 lacI*$^q$-$P_{tac}$ | **CCGCCAAAACAGCCAAGCTT**TTATACGTTTCGGTGCATGCTACTCT |
| RLS31 | Fwd_*ABUW_RS18490 lacI*$^q$-$P_{tac}$ | **TTTAAGAAGGAGATATACAT**ATGAACAACAATATTATAATTTTTGGTTATGGAACA |
| RLS32 | Rev_*ABUW_RS18490 lacI*$^q$-$P_{tac}$ | **CCGCCAAAACAGCCAAGCTT**TTAAGCAGCTTGAATGGCTTCTC |
| RLS90 | Fwd_*dadA-dadX lacI*$^q$-$P_{tac}$ | **TTTAAGAAGGAGATATACAT**ATGCGCGTAATTGTACTAGGTAGCGG |
| RLS91 | Rev_*dadA-dadX lacI*$^q$-$P_{tac}$ | **CCAAGCTTGCATGCCTGCAG**TTATACCTGATTAATAAATTGAACACGGGCGGT |
| RLS93 | Fwd _*ABUW_RS18440 lacI*$^q$*Ptac* | **TTTAAGAAGGAGATATACATAT**GTCTAATTCAGATATACAAAAGATTAACACTAACGAAG |
| RLS92 | Rev_*ABUW_RS18440 lacI*$^q$-*Ptac* | **CCAAGCTTGCATGCCTGCAG**TTACTTTTGTGCTGCCGTCACGG |
| RLS107 | Fwd_*cycA2 lacI*$^q$-$P_{tac}$ | **TTTAAGAAGGAGATATACAT**ATGACAACGGCTCGTCATTCAGATACG |
| RLS108 | Rev_*cycA2 lacI*$^q$-$P_{tac}$ | **CCAAGCTTGCATGCCTGCAGT**TAATGGTCGTTGACGAGTTCACGTCC |
| RLS183 | Rev_*dadA lacI*$^q$-$P_{tac}$ | **AACAGCCAAGCTTGCATGCCTGCAG**TTAAGCCGCATGTGCGTGTGAAT |
| RLS184 | Fwd_*dadX lacI*$^q$-$P_{tac}$ | **AATTCTTTAAGAAGGAGATATACAT**ATGCCTCGTCCTATTACCGCAGT |
| RLS185 | Fwd_*cycA1 lacI*$^q$-$P_{tac}$ | **TTTAAGAAGGAGATATACAT**ATGACAATGGTTCATCATTCAGATGAGGCAAG |
| RLS186 | Rev_*cycA1 lacI*$^q$-$P_{tac}$ | **CCAAGCTTGCATGCCTGCAGT**TAAAAGATTTCTTGACCTAATTTTCTCATACGCGG |

| ***Primers for qPCR*** | | |
|---|---|---|
| RLS35 | Fwd_*recA* | CGCAAACTTGGTGTAGATATTG |
| RLS36 | Rev_*recA* | TTAAATCAATTGCGCCTGAAC |
| RLS41 | Fwd_*cycA2* | TACTATTTGGGTTGGACCTATTG |
| RLS42 | Rev_*cycA2* | GCCAGAAGCTCAAATAGTTAATG |
| RLS47 | Fwd_*csrB* | GGGAATGGATAATCCCAAGTAA |
| RLS48 | Rev_*csrB* | TTTATCGCTTCCGTTCTAACA |
| RLS233 | Fwd_*dadA* | CTGTGAACAAAGAACGTATGATG |

| | | |
|---|---|---|
| RLS234 | Rev_*dadA* | TGTGCTTCTTTACGGAATAACT |
| RLS352 | Fwd_*lrp* | GAATTGGCATCAAGAGTCAAC |
| RLS535 | Rev_*lrp* | GGGTTTAGACGTGCGTAATA |

**Note:** Bold text indicates homology to the respective plasmid backbone, underlined text indicates an added mutation.

**Dataset S1 (separate file).** Table containing detailed mutation information for the Δ*csrA* suppressor strains (CS).

**Dataset S2 (separate file).** Genes differentially expressed in the AB5075 Δ*lrp* cells compared to wild-type (WT) AB5075 cells. Genes exhibiting a log-2 fold change greater than 1 (increased expression in Δ*lrp*) or less than -1 (decreased expression in Δ*lrp*) and having an adjusted p-value ≤ 0.05 are reported as determined by DESeq2 analysis.

**SI References**


1. D. G. Gibson *et al.*, Enzymatic assembly of DNA molecules up to several hundred kilobases. *Nature methods* **6**, 343-345 (2009).
2. M. J. Gebhardt *et al.*, Joint Transcriptional Control of Virulence and Resistance to Antibiotic and Environmental Stress in Acinetobacter baumannii. *mBio* **6**, e01660-01615 (2015).
3. V. Intorcia, R. L. Sava, G. P. Schroeder, M. J. Gebhardt, A series of vectors for inducible gene expression in multidrug-resistant *Acinetobacter baumannii*. *Appl Environ Microbiol* **90**, e0047424 (2024).
4. E. E. Bacon, J. S. Tran, N. Nadig, J. M. Peters, Modular, inducible, and titratable expression systems for *Escherichia coli* and *Acinetobacter baumannii*. *Microbiol Spectr* **12**, e0130624 (2024).
5. M. J. Gebhardt *et al.*, GigC, a LysR Family Transcription Regulator, Is Required for Cysteine Metabolism and Virulence in *Acinetobacter baumannii*. *Infection and immunity* **89**, e00180-00120 (2020).
6. G. Baisa, N. J. Stabo, R. A. Welch, Characterization of *Escherichia coli* D-Cycloserine Transport and Resistant Mutants. *Journal of Bacteriology* **195**, 1389-1399 (2013).
7. K. M. Hujer *et al.*, Analysis of Antibiotic Resistance Genes in Multidrug-Resistant *Acinetobacter* sp. Isolates from Military and Civilian Patients Treated at the Walter Reed Army Medical Center. *Antimicrobial Agents and Chemotherapy* **50**, 4114-4123 (2006).
8. L. Poirel, O. Menuteau, N. Agoli, C. Cattoen, P. Nordmann, Outbreak of extendedspectrum beta-lactamase VEB-1-producing isolates of *Acinetobacter baumannii* in a French hospital. *Journal of clinical microbiology* **41**, 3542-3547 (2003).
9. B. Longo *et al.*, Molecular findings and antibiotic-resistance in an outbreak of *Acinetobacter baumannii* in an intensive care unit. *Ann Ist Super Sanita* **43**, 83-88 (2007).
10. C. La Forgia *et al.*, Management of a multidrug-resistant *Acinetobacter baumannii* outbreak in an intensive care unit using novel environmental disinfection: a 38-month report. *American journal of infection control* **38**, 259-263 (2010).
11. A. C. Jacobs *et al.*, AB5075, a Highly Virulent Isolate of *Acinetobacter baumannii*, as a Model Strain for the Evaluation of Pathogenesis and Antimicrobial Treatments. *MBio* **5**, e01076-01014 (2014).
12. V. Intorcia, M. N. Daum, B. Cheng, S. L. Dove, M. J. Gebhardt, Hfq orchestrates a robust RNA-RNA interaction network in A*cinetobacter baumannii*. *mBio* **17**, e0323125 (2026).
13. L. A. Gallagher *et al.*, Resources for Genetic and Genomic Analysis of Emerging Pathogen *Acinetobacter baumannii*. *J Bacteriol* **197**, 2027-2035 (2015).
